# Pan-cancer analysis of phagocytosis regulators in female-specific cancers: a focus on HMGB2

**DOI:** 10.3389/fimmu.2025.1565924

**Published:** 2025-05-06

**Authors:** Xiaoqin Lu, Dan Ren, Panpan Zhao, Yanfang Li, Zhenhui Wang, Jingyan Zhang

**Affiliations:** Department of Obstetrics and Gynecology, The Second Affiliated Hospital of Zhengzhou University, Zhengzhou, Henan, China

**Keywords:** phagocytosis regulators, HMGB2, female-specific cancers, tumor microenvironment, cancer proliferation and invasion, therapeutic targets

## Abstract

**Introduction:**

Tumor-associated macrophages (TAMs) play a crucial role in the tumor microenvironment, regulating immune escape and promoting cancer progression. Understanding the role of phagocytosis regulators in female-specific cancers is essential for developing effective therapeutic strategies.

**Methods:**

We performed comprehensive analyses of public databases to evaluate the expression, somatic mutations, and copy number variations of phagocytosis regulators. DNA methylation patterns, biological pathways, survival outcomes, and drug sensitivity were assessed. Additionally, immune modulators, immune cell infiltration, and single-cell sequencing were used to explore alterations in phagocytosis and their cellular origins. The functional role of HMGB2 in tumor cell behavior was validated through *in vitro* assays.

**Results:**

Phagocytosis regulators exhibited differential expression across various female-specific cancers, with key genes such as CD47 and FOXO1 playing significant roles in modulating tumor progression. High-frequency mutations were found in PTEN, ARID1A, and UBR4. Genes like COX5B and MS4A1 emerged as potential predictors of clinical outcomes and therapeutic response. HMGB2 knockdown significantly inhibited cancer cell proliferation, migration, and invasion in female-specific cancers. HMGB2 knockdown in macrophages led to a significant impairment in phagocytosis of breast, cervical, ovarian, and endometrial cancer cells. Furthermore, when HMGB2 knockdown was combined with Palbociclib treatment, a significant decrease in tumor cell proliferation was observed across multiple cancer models.

**Conclusion:**

This study highlights the pivotal role of phagocytosis regulators, particularly HMGB2, in the progression of female-specific cancers. Targeting HMGB2 offers promising therapeutic opportunities, potentially enhancing precision oncology and improving patient outcomes.

## 1 Introduction

Female-specific cancers, particularly breast, cervical, ovarian, and endometrial cancers, collectively represent a critical health concern globally due to their high incidence and significant impact on mortality rates. Breast cancer remains the most frequently diagnosed malignancy in women worldwide, with an estimated 2.3 million new cases in 2020 alone, surpassing even lung cancer as the leading cause of cancer-related deaths among women (1). Cervical cancer, though largely preventable through HPV vaccination and screening, still contributes substantially to cancer mortality, especially in low-resource settings where access to preventative care is limited (2). Ovarian cancer, often diagnosed at an advanced stage due to its asymptomatic nature in early phases, carries a poor prognosis, with five-year survival rates under 50% in many regions, necessitating advances in early detection strategies (3). Endometrial cancer, while generally detected at earlier stages, has been rising in incidence, partly attributed to increasing obesity rates and hormonal changes, emphasizing the importance of preventative measures and awareness (4).

Collectively, these cancers account for a significant portion of the cancer burden among women, affecting not only survival but also overall quality of life and placing a considerable strain on healthcare systems globally (5). The exploration of molecular mechanisms underlying these malignancies is crucial, as understanding these pathways can guide the development of novel therapeutic strategies and improve patient outcomes.

The pan-cancer research approach, which examines patterns across multiple cancer types to identify common molecular mechanisms, has emerged as a powerful strategy to understand tumorigenesis on a broader scale (6, 7). By investigating molecular alterations shared among various cancers, pan-cancer studies enable the discovery of universal targets that can inform both diagnostics and therapeutics, potentially offering new insights into pathways that are consistently dysregulated in cancer (8–10). A critical area of interest in pan-cancer analysis is the immune response, particularly mechanisms that tumors exploit to evade immune surveillance, such as disruptions in phagocytosis pathways (11).

Phagocytosis, a key process within the innate immune response, is essential for the elimination of pathogens and abnormal cells, including cancer cells. However, tumors can develop strategies to evade this defense mechanism, thus avoiding immune destruction and promoting tumor growth and progression (12). Specific phagocytosis regulatory factors, such as the “don’t eat me” signals, including CD47, allow cancer cells to inhibit macrophage-mediated phagocytosis, facilitating immune escape and enhancing metastatic potential (13). Tumors often exploit mechanisms to evade phagocytosis, notably through the CD47-SIRPα axis (14). The interaction between CD47, overexpressed on many cancer cells, and SIRPα on phagocytes delivers a “don’t eat me” signal, inhibiting phagocytosis and facilitating immune evasion (15). Targeting this pathway has emerged as a promising therapeutic strategy, as disrupting the CD47-SIRPα interaction enhances phagocytic activity and activates both innate and adaptive immune responses against tumors (15). Additionally, tumor-associated macrophages (TAMs) can adopt immune-suppressive phenotypes, secreting cytokines that inhibit effector immune cells and promote tumor progression (16). Furthermore, TAMs can influence the tumor microenvironment by altering extracellular matrix composition, which may enhance tumor cell survival and invasion. Therefore, understanding and modulating phagocytic pathways, including the CD47-SIRPα axis and TAM behavior, holds significant potential for improving cancer immunotherapy outcomes (17). Targeting these mechanisms could lead to more effective treatments, particularly in cancers where immune evasion is a significant hurdle.

The primary objective of this study is to systematically analyze the expression patterns, genomic alterations, DNA methylation, and associated biological pathways of phagocytosis regulatory factors across female-specific cancers, including breast, cervical, ovarian, and endometrial cancers. Furthermore, this research aims to explore the relationship between these regulators and the tumor immune microenvironment, immune modulators, drug sensitivity, and key clinical characteristics. Experimental validation was conducted to assess the functional impact of selected key genes on tumor cell behaviors using *in vitro* models. Ultimately, the study emphasizes the translational potential of phagocytosis regulatory factors as biomarkers and therapeutic targets, offering insights into their clinical utility for improving precision medicine strategies in female-specific cancers.

## 2 Materials and methods

### 2.1 Analysis of phagocytosis regulator expression

Clinical information and genomic data for breast invasive carcinoma (BRCA), cervical squamous cell carcinoma and endocervical adenocarcinoma (CESC), ovarian serous cystadenocarcinoma (OV), and uterine corpus endometrial carcinoma (UCEC) were obtained from The Cancer Genome Atlas (TCGA) via the UCSC Xena platform (https://xena.ucsc.edu/welcome-to-ucsc-xena/). For each cancer type, adjacent normal tissue samples served as paracancerous controls, while tumor samples represented the disease group. Differential expression analysis was conducted using the R package “limma,” with phagocytosis regulators considered differentially expressed if |log2FC| > 1 and FDR < 0.05 between tumor and paracancerous samples.

### 2.2 Analysis of somatic mutation and copy number variation data

A total of 89 phagocytosis regulators were selected based on previous studies13. The average expression of these regulators in each sample was calculated to generate a “phagocytic score,” providing an overall estimate of phagocytic regulator expression levels. Somatic mutation data and copy number variation (CNV) data for female-specific cancers were sourced from the TCGA database (http://gdac.broadinstitute.org/).

### 2.3 DNA methylation analysis

DNA methylation data for female-specific cancers were obtained from the TCGA database (http://gdac.broadinstitute.org/). To assess the impact of promoter DNA methylation on the expression of phagocytosis regulators, we focused on methylation sites within the transcription start site region, spanning −1,500 bp to +500 bp. Promoter DNA methylation disorder was defined as sites showing a Beta value change > 0.2 between normal and tumor samples, with an FDR < 0.05. Spearman correlation analysis was performed between these methylation sites and the expression of phagocytosis regulators, retaining only the site with the smallest Spearman correlation coefficient.

### 2.4 Biological pathway analysis

We identified 10 frequently altered signaling pathways from a previous study17, including the HIPPO, NOTCH, PI3K, RTK/RAS, TGF-Beta, TP53, WNT, Cell Cycle, NRF2, and MYC pathways. Pathway scores were calculated using single-sample gene set enrichment analysis (ssGSEA), followed by Spearman correlation analysis between phagocytosis regulators and pathway scores. Phagocytosis regulators were considered to be associated with these pathways if they had a corrected P value < 0.05 and an absolute correlation value > 0.3.

### 2.5 Survival analysis

Patients were stratified into high and low expression groups based on the median expression levels of phagocytosis regulators. The Kaplan–Meier (KM) overall survival curve was generated using the survival and Survminer R packages, and the log-rank test was applied to assess the statistical significance of survival differences between high and low expression groups. Univariate Cox regression analysis was used to evaluate the impact of each phagocytosis regulator on cancer prognosis.

### 2.6 Drug sensitivity analysis

Data from the Genomics of Drug Sensitivity in Cancer (GDSC) database (http://www.cancerrxgene.org/downloads) were downloaded for the GDSC2 cohort, which includes standardized gene expression profiles for 805 cancer cell lines along with IC50 values for 198 small-molecule drugs. Spearman correlation analysis was conducted between the expression of phagocytosis regulators and the IC50 values of these drugs. A significance threshold was set at an absolute correlation value ≥ 0.3 with P < 0.05.

### 2.7 Immunomodulators and immune cell infiltration analysis

Immunomodulators encompass a group of immune regulatory genes, including antigen-presenting molecules, ligands, and receptors, which are critical in cancer immunotherapy. We utilized a list of immune modulators provided by the TCGA immune response working group18. Immune cell infiltration scores in female cancers were calculated using ssGSEA, and Spearman correlation analysis was employed to assess associations between the phagocytic score, immune cell infiltration scores, and immune modulators. A corrected P value < 0.05 was set as the threshold for significance.

### 2.8 Cell source of phagocytosis alterations

Single-cell sequencing datasets for breast and ovarian cancer were obtained from a previous study19, and data were downloaded from https://lambrechtslab.sites.vib.be/en/pan-cancer-blueprint-tumour-microenvironment-0. Single-cell sequencing data for cervical cancer and endometrial cancer were acquired from the GEO database (https://www.ncbi.nlm.nih.gov/gds) under accession numbers GSE168652 and GSE173682, respectively20,21.

Pre-processing of single-cell RNA sequencing (scRNA-seq) data was conducted using the Seurat package (version 4.0.0) in R. The “NormalizeData” function was applied to standardize the scRNA-seq data, and 2,000 highly variable genes were identified using the “FindVariableFeatures” function. To correct for batch effects across different sample sources, batch correction was performed using the Harmony package in R. Following batch correction, data scaling was performed, and dimensionality reduction was achieved through principal component analysis (PCA), selecting the top 20 principal components for downstream analysis. The t-SNE algorithm was employed for data visualization and analysis. Cell clusters were identified using the k-nearest neighbor algorithm with the “FindClusters” function at a resolution of 0.2. Finally, cell populations were annotated, and expression levels of phagocytosis regulators were compared across different cell populations.

### 2.9 Cell culture

The breast cancer cell lines (MDA-MB-231, T-47D), cervical cancer cell lines (HeLa, SiHa), ovarian cancer cell lines (SK-OV-3, OVCAR3) and endometrial cancer cell lines (RL95-2, Ishikawa) used in this study were purchased from Procell Life Science & Technology Co., Ltd. (Wuhan, China). All cell lines used in this study were cultured under standard conditions at 37°C in a humidified incubator with 5% CO_2_. The details of cell culture conditions for each cell line are as follows:

MDA-MB-231 cells were cultured in high-glucose DMEM medium (Procell, PM150210, China) supplemented with 10% FBS (Gibco, 164210-50, China) and 1% P/S (CELL RESEARCH, CSP006, China). T-47D cells were cultured in RPMI-1640 medium (Procell, PM150110, China) supplemented with 10% FBS (Gibco, 164210-50, China), 1% P/S (CELL RESEARCH, CSP006, China), and 10 μg/mL insulin (CELL RESEARCH, CSP001-10, China).

HeLa and SiHa cells were cultured in MEM medium containing NEAA (Procell, PM150410, China) supplemented with 10% fetal bovine serum (FBS; Gibco, 164210-50, China) and 1% penicillin-streptomycin solution (P/S; CELL RESEARCH, CSP006, China).

SK-OV-3 cells were cultured in McCoy’s 5A medium (Procell, PM150710, China) supplemented with 10% FBS (Gibco, 164210-50, China) and 1% P/S (CELL RESEARCH, CSP006, China). OVCAR3 cells were cultured in RPMI-1640 medium (Procell, PM150110, China) supplemented with 20% FBS (Gibco, 164210-50, China), 1% P/S (CELL RESEARCH, CSP006, China), and 10 μg/mL insulin (CELL RESEARCH, CSP001-10, China).

RL95–2 cells were cultured in DMEM/F12 medium (Procell, PM150312, China) supplemented with 10% FBS (Gibco, 164210-50, China), 1% P/S (CELL RESEARCH, CSP006, China), and 5 μg/mL insulin (CELL RESEARCH, CSP001-10, China). Ishikawa cells were cultured in high-glucose DMEM medium (Procell, PM150210, China) supplemented with 10% FBS (Gibco, 164210-50, China) and 1% P/S (CELL RESEARCH, CSP006, China).

### 2.10 Cell counting kit-8 assay

Cell proliferation was assessed using the CCK8 according to the manufacturer’s protocol. Briefly, cells were seeded into 96-well plates at a density of 2×10^3^cells per well in 100 μL of complete medium and allowed to adhere overnight. At designated time points (24, 48, 72, and 96 hours), 10 μL of CCK-8 reagent was added to each well and incubated at 37°C for 2 hours. The absorbance was measured at 450 nm using a microplate reader. Each experiment was performed in triplicate, and results were expressed as the mean ± standard deviation (SD).

### 2.11 5-Ethynyl-2’-deoxyuridine assay

Cells were seeded in 96-well plates, treated, and then incubated with 10 µM EdU labeling medium for 2 hours. Following fixation with 4% paraformaldehyde and permeabilization with 0.5% Triton X-100, cells were treated with the EdU reaction cocktail according to the manufacturer’s instructions. Images were captured using a fluorescence microscope, and EdU-positive cells were quantified to determine proliferation rates.

### 2.12 Transwell assay for migration and invasion

To assess cell migration and invasion, Transwell chambers were used. For migration assays, cells were seeded into the upper chamber with serum-free medium, while the lower chamber contained medium with 10% FBS as a chemoattractant. For invasion assays, the upper chamber was pre-coated with Matrigel. After incubation for 24 hours at 37°C, non-migrated or non-invaded cells were removed, and cells on the lower membrane surface were fixed, stained, and counted under a microscope.

### 2.13 Fluorescence microscopy-based phagocytosis assay

To assess macrophage-mediated phagocytosis, we co-cultured RAW264.7 macrophages, with and without HMGB2 knockdown, with tumor cells (MDA-MB-231, HeLa, SKOV3, and Ishikawa) that were pre-labeled with PHRODO RED dye. The cells were incubated for 2 hours to allow phagocytosis. After washing away non-phagocytosed tumor cells, macrophages were fixed and stained for nuclear visualization. Fluorescent images were captured using microscope. The phagocytic index was calculated by counting the tumor cells engulfed by macrophages in randomly selected fields.

### 2.14 Statistical analysis

All statistical analyses were performed using R software version 4.2.0 (http://www.r-project.org). Correlation analyses were conducted using the Spearman correlation test. Survival risk and hazard ratios were calculated with the Cox proportional hazards model. The Kaplan–Meier (KM) survival curve was employed to assess the prognostic significance of each variable, and significance was tested using the log-rank test. For comparisons of various metrics (expression level, infiltration ratio, and other characteristic values), the Wilcoxon rank-sum test was applied for two-group comparisons, while the Kruskal–Wallis test was used for comparisons across multiple groups. A statistical significance threshold was set at P < 0.05.

## 3 Results

### 3.1 Expression of phagocytosis regulators in female cancers

**Figure 1:**
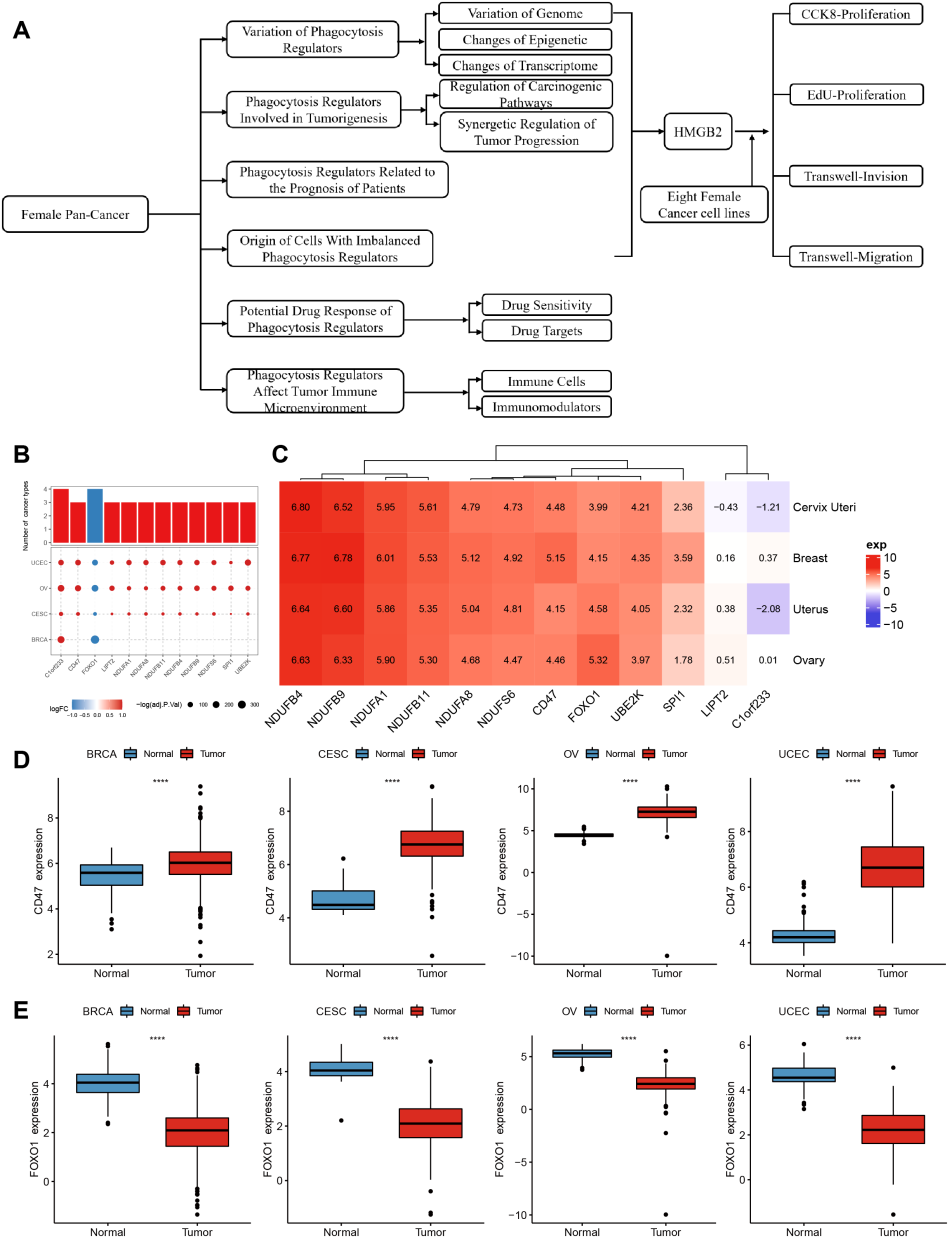
Differential expression and distribution of phagocytosis regulators across multiple female cancers. **(A)** flow chart. **(B)** Phagocytosis regulator expression differed in at least three cancers. **(C)** A heatmap displaying the average expression levels of significantly dysregulated phagocytosis regulators across four cancer types. Genes such as NDUFB4, CD47, and FOXO1 show distinct expression patterns, highlighting their potential roles in tumor biology. Positive values (red) indicate higher expression, while negative values (blue) represent lower expression in tumor tissues compared to normal tissues. **(D)** Box plots illustrating the expression levels of CD47 in tumor and normal samples across BRCA, CESC, OV, and UCEC. CD47 expression is significantly higher in tumor tissues compared to normal tissues across all four cancer types. **(E)** Box plots showing the expression levels of FOXO1 in tumor and normal samples across BRCA, CESC, OV, and UCEC. FOXO1 expression is significantly lower in tumor tissues compared to normal tissues across all four cancers. ****p < 0.0001.

The expression profiles of 89 phagocytosis regulators in female cancers were analyzed using the limma package, with differential expression defined as |log2FC| > 1 and FDR < 0.05. The results of this analysis are summarized in **Table 1**. Twelve phagocytosis regulators were identified as having significant expression changes in at least three cancer types, as illustrated in **Figure 1A**. Notably, CD47 demonstrated consistently high expression across all four cancer types, whereas forkhead box O1 (FOXO1) exhibited significantly reduced expression in these cancers (**Figures 1B, C**). Furthermore, the average expression levels of these differentially expressed regulators were evaluated in various normal tissues using data from the GTEx database. Among the identified genes, C1orf233 stood out for its relatively lower expression in normal tissues compared to other genes (**Figure 1D**).

**Table 1:** Differential expression of phagocytosis regulators.

Cancer	Up	Down
BRCA	3	1
CESC	15	2
OV	28	7
UCEC	26	5

### 3.2 Genomic variation of phagocytosis regulators in female cancers

**Figure 2:**
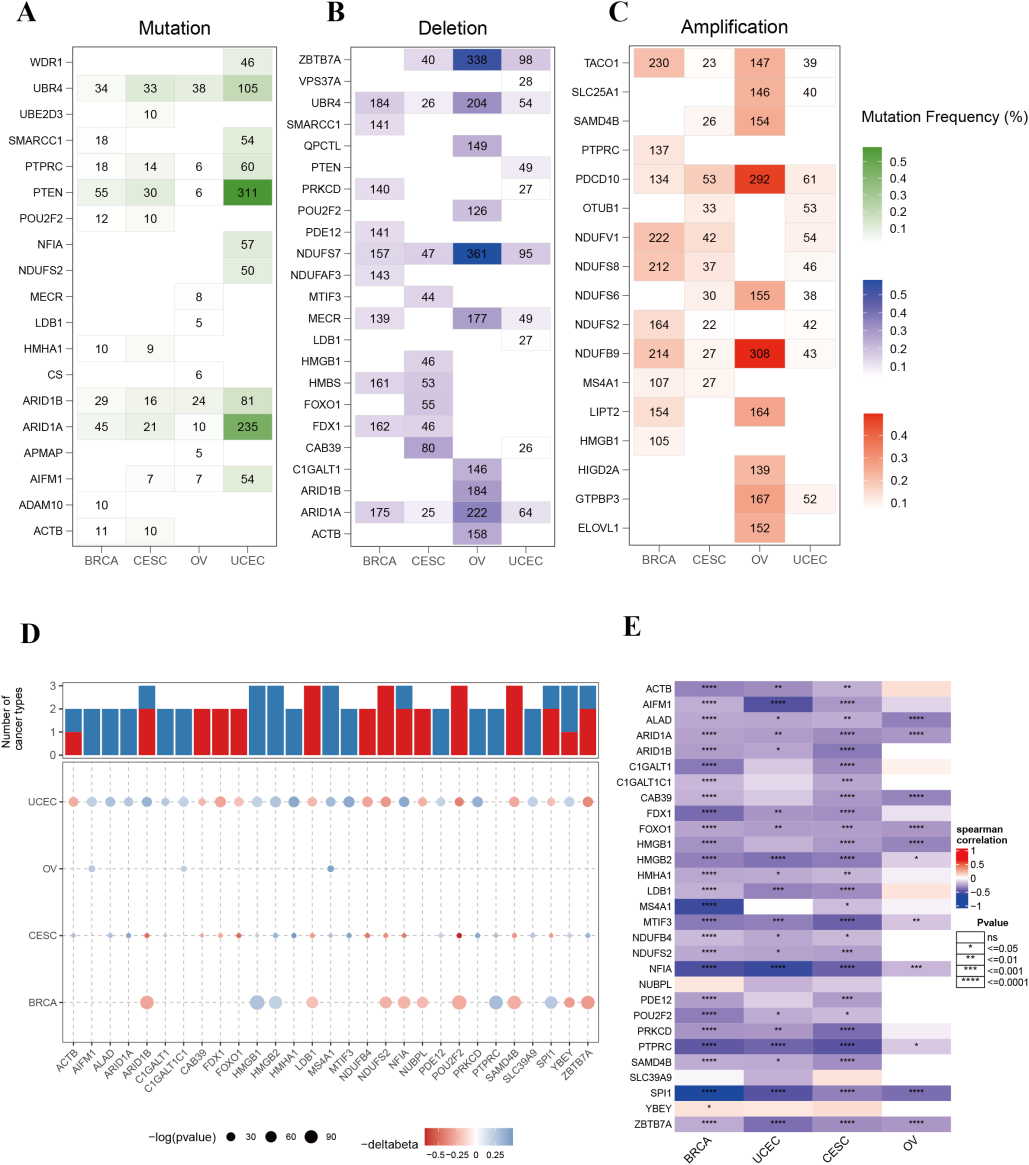
Genomic alterations and epigenetic regulation of phagocytosis regulators in female cancers. **(A)** Mutation frequencies of phagocytosis regulators across four cancer types: breast cancer (BRCA), cervical squamous cell carcinoma (CESC), ovarian cancer (OV), and uterine corpus endometrial carcinoma (UCEC). **(B)** Frequency of copy number deletions for phagocytosis regulators across the four cancer types. **(C)** Frequency of copy number amplifications for phagocytosis regulators across the four cancer types. **(D)** Bar plot and bubble plot illustrating the number of cancers showing significant DNA methylation changes for phagocytosis regulators. Red bars represent hypermethylation, and blue bars represent hypomethylation. Bubble size indicates the statistical significance (-log10 p-value), and color indicates changes in methylation level (Δbeta). **(E)** Heatmap showing the Spearman correlation between DNA methylation and gene expression of phagocytosis regulators in BRCA, UCEC, CESC, and OV. Correlations range from strong negative to strong positive, with statistical significance indicated. *p < 0.05, **p < 0.01, ***p < 0.001, ****p < 0.0001.

To investigate the genomic variations of phagocytosis regulators in female cancers, we analyzed the mutation frequencies of copy number variations (CNVs; including amplifications and deletions) and single-nucleotide variants (SNVs; non-silent mutations) in a pan-cancer cohort encompassing four types of cancer. **Figure 2** highlights the top 10 genes with the highest mutation frequencies in each cancer type. Among these, phosphatase and tensin homolog (PTEN), AT-rich interaction domain 1A (ARID1A), and ubiquitin protein ligase E3 component n-recognin 4 (UBR4) exhibited the highest frequencies of SNV mutations across the cancers analyzed. Notably, PTEN and ARID1A mutations were significantly more frequent in endometrial cancer compared to the other three cancer types (**Figure 2A**). Analysis of CNVs among phagocytosis regulators further revealed distinct patterns across cancers. For example, genes such as programmed cell death 10 (PDCD10) and NADH: ubiquinone oxidoreductase subunit B9 (NDUFB9) displayed increased copy numbers in ovarian cancer, while NDUFS7 and zinc finger and BTB domain-containing 7A (ZBTB7A) exhibited prominent copy number losses in ovarian cancer (**Figures 2B, C**).

### 3.3 Epigenetic changes of phagocytosis regulators in female cancers

DNA methylation serves as a critical regulator of gene expression in cancer. To explore the epigenetic landscape of phagocytosis regulators in female cancers, we analyzed the promoter DNA methylation patterns of these genes across multiple cancer types (**Supplementary Table 1**). A total of 29 phagocytosis regulators were identified with altered methylation levels in at least two cancer types. Importantly, these regulators demonstrated consistent methylation trends across different cancers. For example, high mobility group box 1 (HMGB1), HMGB2, and MS4A1 exhibited hypomethylation, whereas LIM domain binding 1 (LDB1), NDUFS2, and POU class 2 homeobox 2 (POU2F2) displayed hypermethylation (**Figure 2D**). Moreover, a negative correlation was observed between promoter DNA methylation levels and the expression of phagocytosis regulators in female cancers (**Figure 2E**). These results suggest that promoter DNA methylation may play a pivotal role in regulating the expression of phagocytosis regulators, contributing to their functional modulation in these cancers.

### 3.4 Effects of phagocytosis regulators on biological pathways

**Figure 3:**
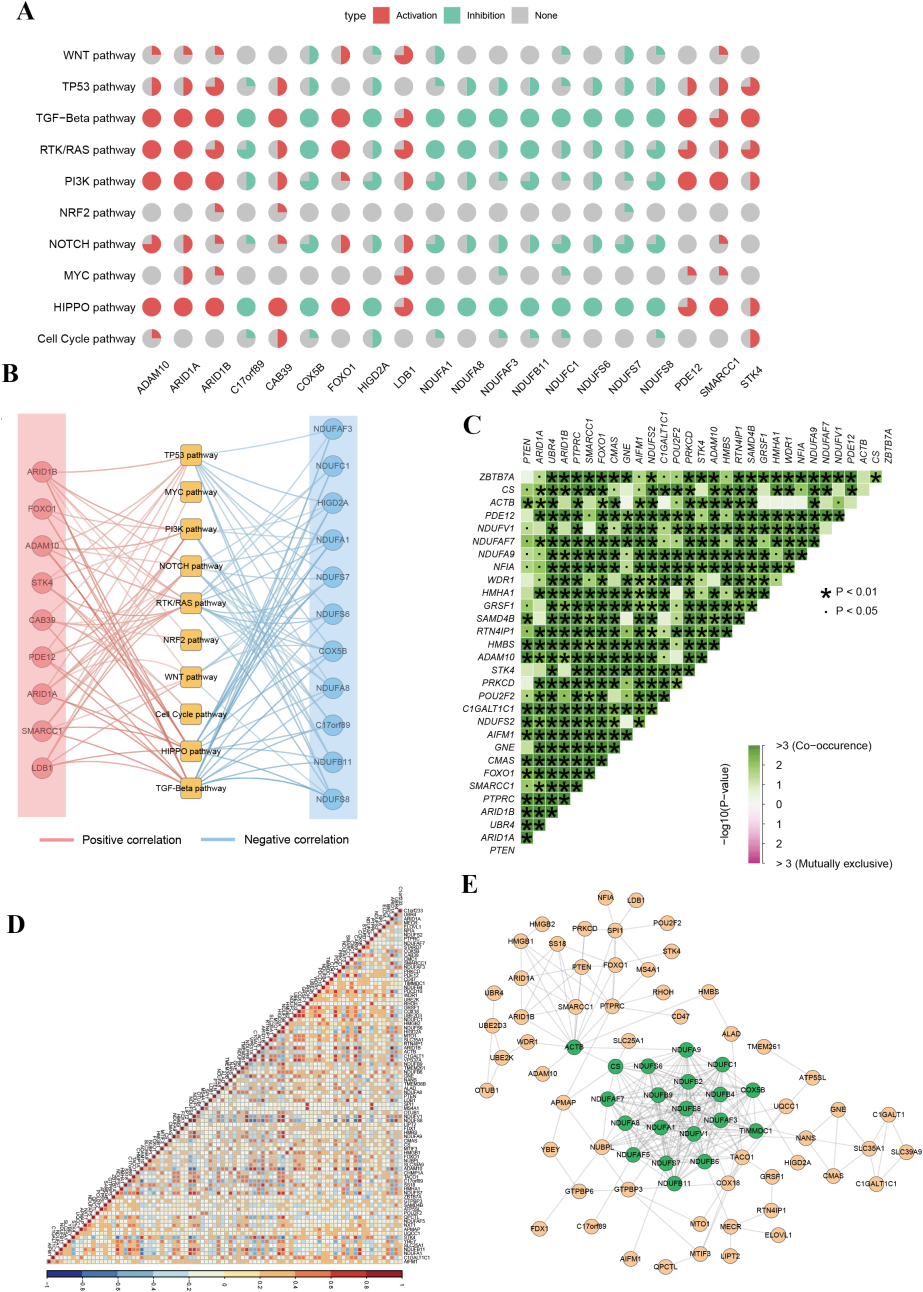
Functional pathway analysis and co-regulation networks of phagocytosis regulators in female cancers. **(A)** Pie chart summary illustrating the impact of phagocytosis regulators on key signaling pathways across female cancers. **(B)** Network representation of the correlation between phagocytosis regulators and signaling pathways. Red edges indicate positive correlations, and blue edges represent negative correlations. **(C)** Co-occurrence and mutual exclusivity analysis of phagocytosis regulators. A heatmap displays co-occurrence relationships (green) and statistical significance (p-values < 0.05 or < 0.01). **(D)** Correlation matrix of phagocytosis regulator expression across samples. The heatmap indicates positive (red) or negative (blue) correlations between genes, with high correlation clusters suggesting coordinated regulatory mechanisms. **(E)** Protein-protein interaction network of phagocytosis regulators.

To elucidate the molecular mechanisms through which phagocytosis regulators contribute to tumorigenesis, we analyzed their involvement in 10 key signaling pathways using the ssGSEA algorithm. Spearman correlation analysis was performed to assess the relationship between individual phagocytosis regulators and pathway activity scores. The top 20 regulators with the highest number of pathway correlations are presented in **Figure 3** and **Supplementary Table 2**. The results revealed that phagocytosis regulators are significantly associated with either the activation or inhibition of various oncogenic pathways. For instance, ADAM metallopeptidase domain 10 (ADAM10) and ARID1A showed positive correlations with the activation of pathways such as HIPPO, PI3K, RTK/RAS, and TGF-Beta, whereas COX5B and NDUFA1 were associated with the inhibition of pathways including HIPPO, NOTCH, and PI3K (**Figure 3A**). These findings suggest that phagocytosis regulators play a critical role in modulating key carcinogenic pathways in female cancers. Among the four cancer types analyzed, endometrial cancer exhibited the strongest correlations between phagocytosis regulators and signaling pathways, warranting further investigation (**Figure 3B**). To better understand the cooperative functions of these genes, we conducted a co-mutation analysis focusing on phagocytosis regulators with single-nucleotide variant (SNV) mutation frequencies exceeding 3% in endometrial cancer (**Figure 3C**). The analysis uncovered distinct patterns of co-mutation among phagocytosis regulators, suggesting their collective role in activating oncogenic pathways that drive tumor progression. Additionally, correlation analysis revealed an overall positive association among phagocytosis regulators in endometrial cancer (**Figure 3D**). Furthermore, the protein-protein interaction network analysis identified key hub genes, including COX5B, NDUFS8, NDUFB9, and ACTB, which exhibited high degrees of interaction. These hub genes likely play pivotal roles in the phagocytosis regulator network, underscoring their potential significance in tumor progression (**Figure 3E**).

### 3.5 Prognostic value of phagocytosis regulators in female cancer patients

**Figure 4:**
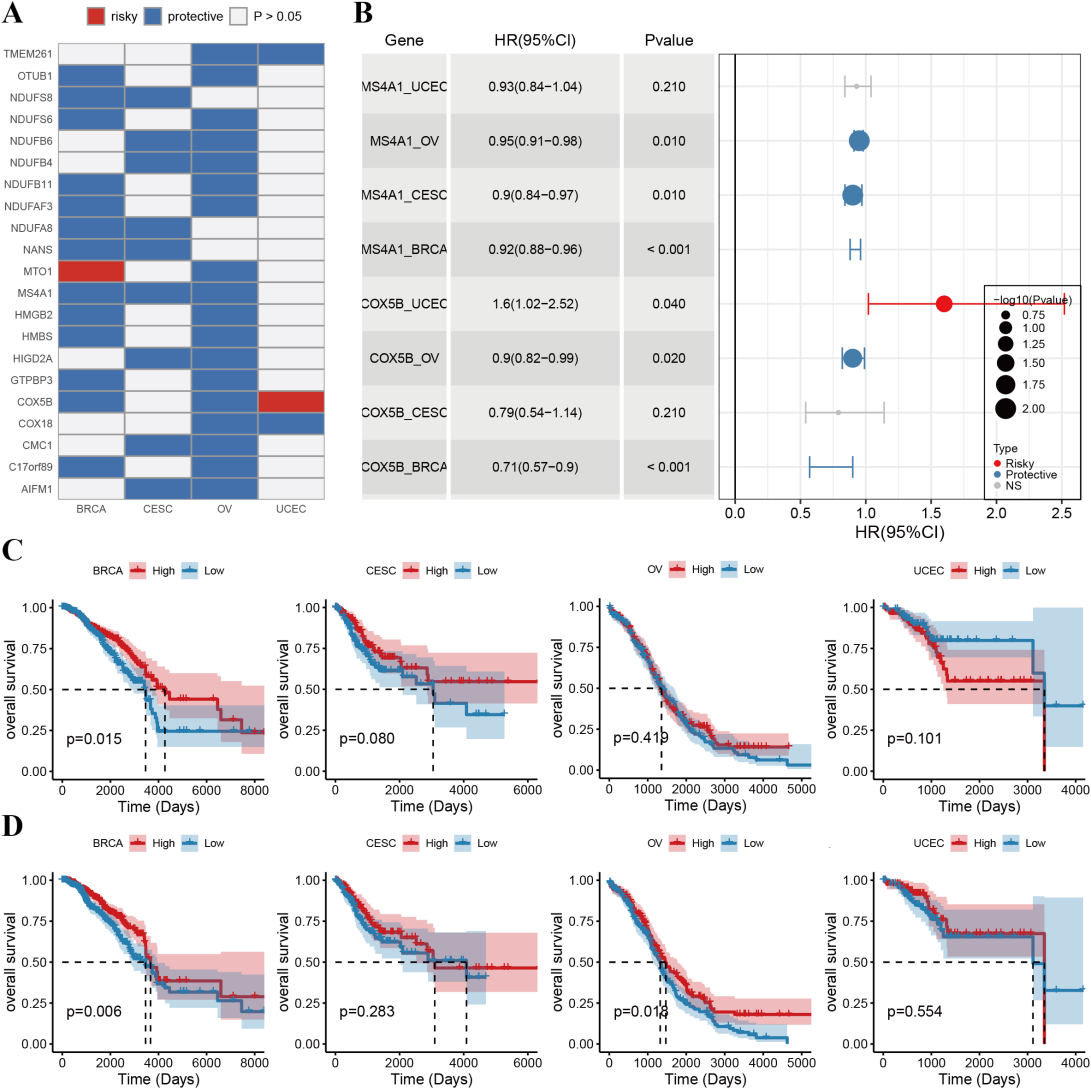
Prognostic value of phagocytosis regulators in female cancers. **(A)** Heatmap showing the prognostic roles of phagocytosis regulators across breast cancer (BRCA), cervical squamous cell carcinoma (CESC), ovarian cancer (OV), and uterine corpus endometrial carcinoma (UCEC). Genes are classified as either risky (red) or protective (blue) factors based on their hazard ratios (HRs). White indicates non-significant (p > 0.05) prognostic results. **(B)** Forest plot summarizing the HRs and 95% confidence intervals (CIs) of key prognostic phagocytosis regulators. The bubble size represents the -log10(p-value), with risky factors shown in red and protective factors in blue. **(C)** Kaplan-Meier survival curves illustrating the overall survival (OS) stratified by the expression levels of COX5B in BRCA, CESC, OV, and UCEC. High expression of COX5B is associated with improved OS in BRCA (p = 0.015) and OV (p = 0.041) but not in CESC or UCEC. **(D)** Kaplan-Meier survival curves for MS4A1 in the same cancers. High expression of MS4A1 is linked to better OS in BRCA (p = 0.006) and OV (p = 0.014), while no significant associations are observed in CESC or UCEC.

Given the critical role of phagocytosis regulators in cancer, we evaluated their prognostic significance in female cancer patients. Single-factor Cox regression analysis was conducted for 89 phagocytosis regulators across various tumor types, focusing on those associated with overall survival in at least two cancer types (**Figure 4**, **Supplementary Table 3**). The analysis identified 21 phagocytosis regulators significantly linked to overall survival across multiple female cancers (**Figure 4A**). Most of these regulators demonstrated protective roles, with their higher expression associated with improved survival. However, exceptions were observed: mitochondrial tRNA translation optimization 1 (MTO1) was identified as a risk factor in breast cancer, while COX5B was associated with increased risk in endometrial cancer. Additionally, COX5B and MS4A1 were notable for their prognostic relevance across three cancer types. The forest plot presents the results of the single-factor Cox regression analysis for COX5B and MS4A1, highlighting their hazard ratios and confidence intervals (**Figure 4B**). Furthermore, Kaplan–Meier (KM) survival curves illustrate the prognostic impact of these genes in different cancer types, underscoring their role as key biomarkers for overall survival (**Figures 4C, D**).

### 3.6 Therapeutic potential of phagocytosis regulators

**Figure 5:**
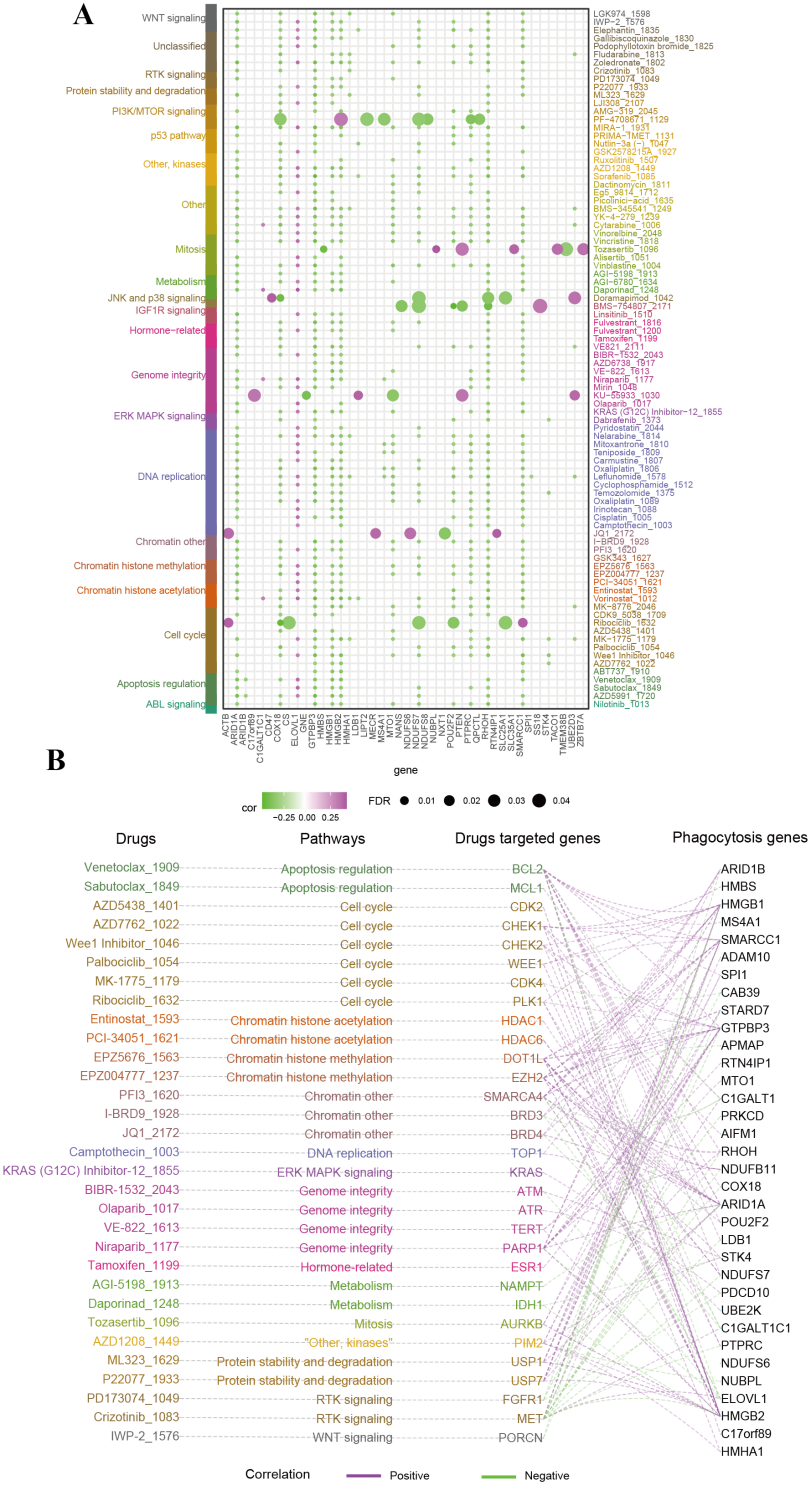
Therapeutic relevance of phagocytosis regulators in female cancers. **(A)** Bubble plot summarizing the correlation between phagocytosis regulators and the sensitivity to small-molecule drugs targeting various pathways. The size of the bubbles represents the false discovery rate (FDR), while the color indicates the correlation coefficient (purple for negative correlation, green for positive correlation). Drugs are categorized based on their associated pathways. Key phagocytosis regulators exhibit significant correlations with drug sensitivity. **(B)** Network diagram linking phagocytosis regulators, drug-targeted genes, and drugs, grouped by pathway.

To investigate the therapeutic potential of phagocytosis regulators in modulating drug responses, we performed a Spearman correlation analysis between the expression of phagocytosis regulators and the sensitivity to small-molecule drugs (**Supplementary Table 4**). This analysis revealed 859 significant correlation pairs between drug sensitivity and phagocytosis regulator expression, with 86 drugs showing associations with at least five phagocytosis regulators (**Figure 5A**). For drugs with well-characterized target genes, we further explored the relationships between these target genes and phagocytosis regulators. For instance, the target gene WEE1 of Palbociclib exhibited positive correlations with multiple phagocytosis regulators, including SMARCC1 (R = 0.30), HMGB2 (R = 0.46), and HMGB1 (R = 0.33). **Figure 5B** illustrates the hierarchical network connecting drug pathways, target genes, and phagocytosis regulators involved in critical metabolic pathways. Given the role of many clinically actionable genes as targets for anti-cancer therapies, these findings suggest that phagocytosis regulators may hold significant therapeutic potential in female-specific cancers, particularly through their impact on drug response and sensitivity.

### 3.7 Effects of phagocytosis regulators on the tumor microenvironment

**Figure 6:**
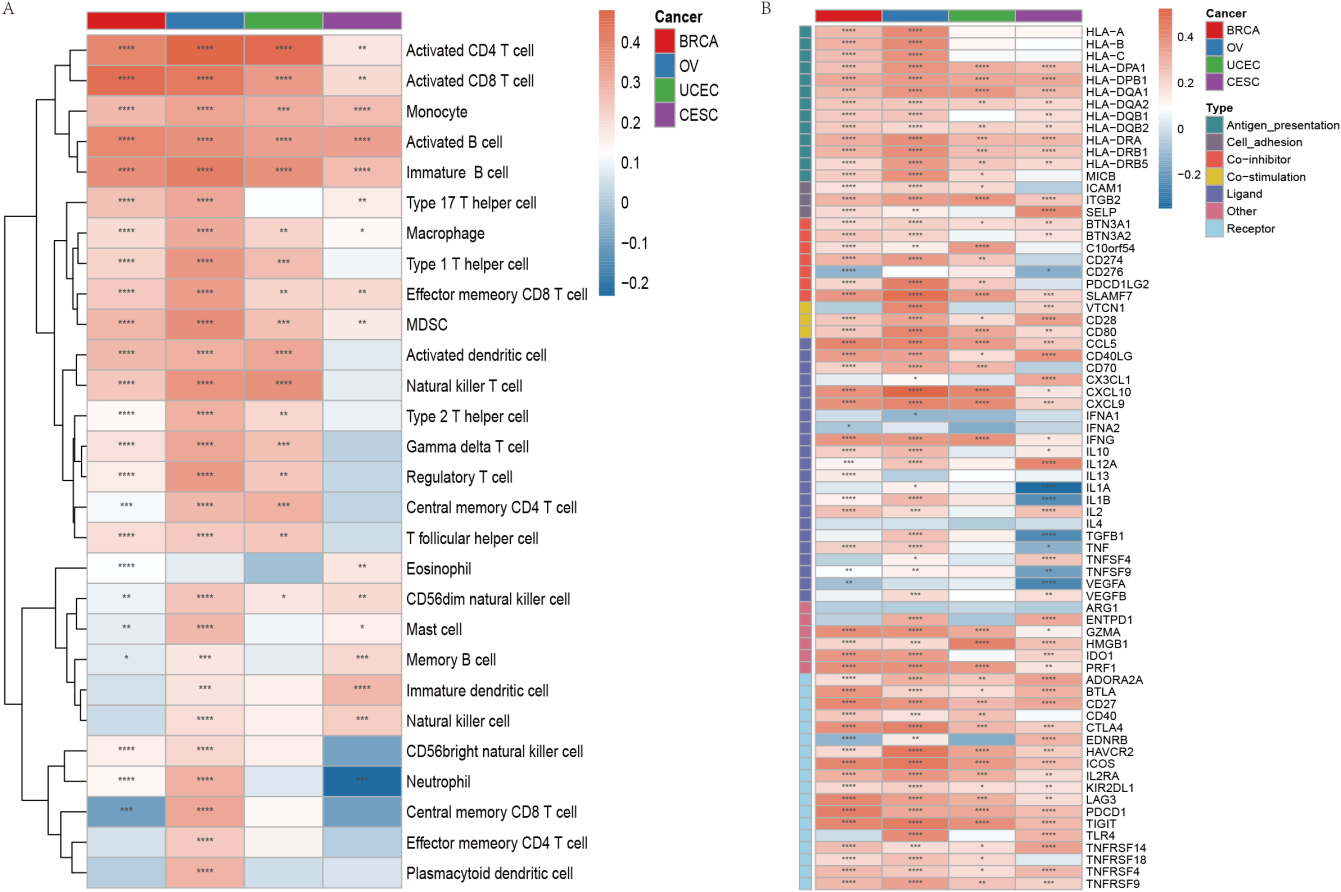
Immune cell infiltration and immunomodulator expression associated with phagocytosis regulators in female cancers. **(A)** Heatmap showing the correlation between the phagocytosis score and the infiltration of various immune cell types across breast cancer (BRCA), ovarian cancer (OV), uterine corpus endometrial carcinoma (UCEC), and cervical squamous cell carcinoma (CESC). The color gradient indicates the Spearman correlation coefficient, with red representing positive correlations and blue representing negative correlations. **(B)** Heatmap illustrating the correlation between the phagocytosis score and key immunomodulators across the four cancer types. Immunomodulators are grouped by their functions. Red indicates positive correlations, and blue indicates negative correlations. *p < 0.05, **p < 0.01, ***p < 0.001, ****p < 0.0001.

Immune cell infiltration is a crucial component of the tumor microenvironment (TME) and plays an essential role in enhancing the efficacy of immunotherapy. To assess the relationship between the phagocytosis score and immune cell infiltration, we quantified immune cell infiltration scores in the TME using the ssGSEA method and calculated the Spearman correlation between the phagocytosis score and immune cell infiltration levels (**Supplementary Table 5**). The results demonstrated a significant positive correlation between the phagocytosis score and the infiltration levels of most immune cells in female cancers (**Figure 6A**). To further investigate the molecular relationship between the phagocytosis score and tumor immunity, we analyzed the Spearman correlation between the phagocytosis score and key immunomodulators involved in immunotherapy. Consistent with our pathway analysis, the phagocytosis score exhibited a positive correlation with various immunomodulators across multiple cancers, suggesting its potential role in regulating tumor immunity (**Figure 6B**).

### 3.8 Cell origins of imbalanced phagocytosis regulators

To explore the cellular origins of dysregulated phagocytosis regulators, we analyzed single-cell RNA sequencing (scRNA-seq) data from breast, cervical, ovarian, and endometrial cancers. Rigorous pre-processing steps were applied, and batch effects were mitigated using the Harmony algorithm. Following normalization and dimensionality reduction via principal component analysis (PCA), we employed the t-distributed Stochastic Neighbor Embedding(t-SNE) method to visualize the data. Using marker genes, we visualized the expression patterns of key genes within each cluster through the “DotPlot” function, enabling the assignment of clusters to specific cell lineages. Furthermore, violin plots were used to illustrate the expression levels of phagocytosis regulators across different cell types, highlighting their distribution within various cell populations.

**Figure 7:**
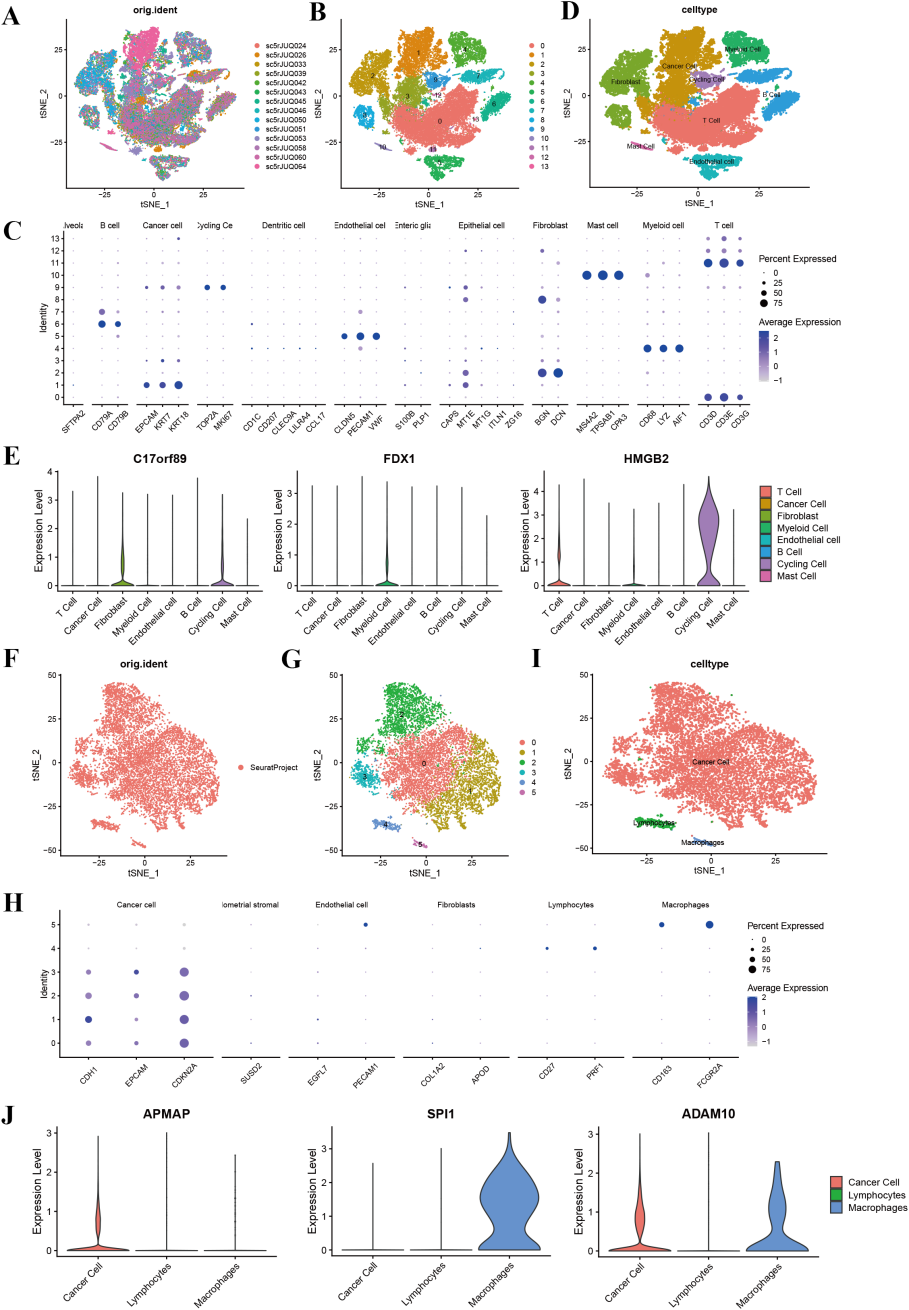
Single-cell sequencing analysis of breast cancer and cervical cancer. **(A)** t-SNE plot showing the distribution of cells across different BRCA samples, with each color representing a unique sample. **(B)** t-SNE plot illustrating clustering of cells into 14 distinct clusters (0–13). **(C)** t-SNE plot with annotated cell types, including cancer cells, fibroblasts, T cells, B cells, myeloid cells, endothelial cells, cycling cells, and mast cells. **(D)** Annotation of 13 clusters into eight different cell types in breast cancer; **(E)** Violin plots displaying the expression levels of key genes (C17orf89, FDX1, and HMGB2) across various cell types in breast cancer. C17orf89 is predominantly expressed in cancer cells and B cells, FDX1 shows the highest expression in Fibroblast cells, and HMGB2 is highly expressed in cancer cells and cycling cells. **(F)** t-SNE plot showing the distribution of cells from different CESC samples, with each color representing a distinct sample. **(G)** t-SNE plot illustrating clustering of cells into six distinct groups (0–5). **(H)** t-SNE plot with annotated cell types, including cancer cells, lymphocytes, and macrophages. **(I)** Annotation of six clusters into three different cell types in cervical cancer. **(J)** Violin plots displaying the expression levels of key genes (APMAP, SPI1, and ADAM10) across the identified cell types. APMAP is predominantly expressed in Lymphocytes, SPI1 is highly expressed in Macrophages, and ADAM10 is primarily expressed in cancer cells.

**Figure 8:**
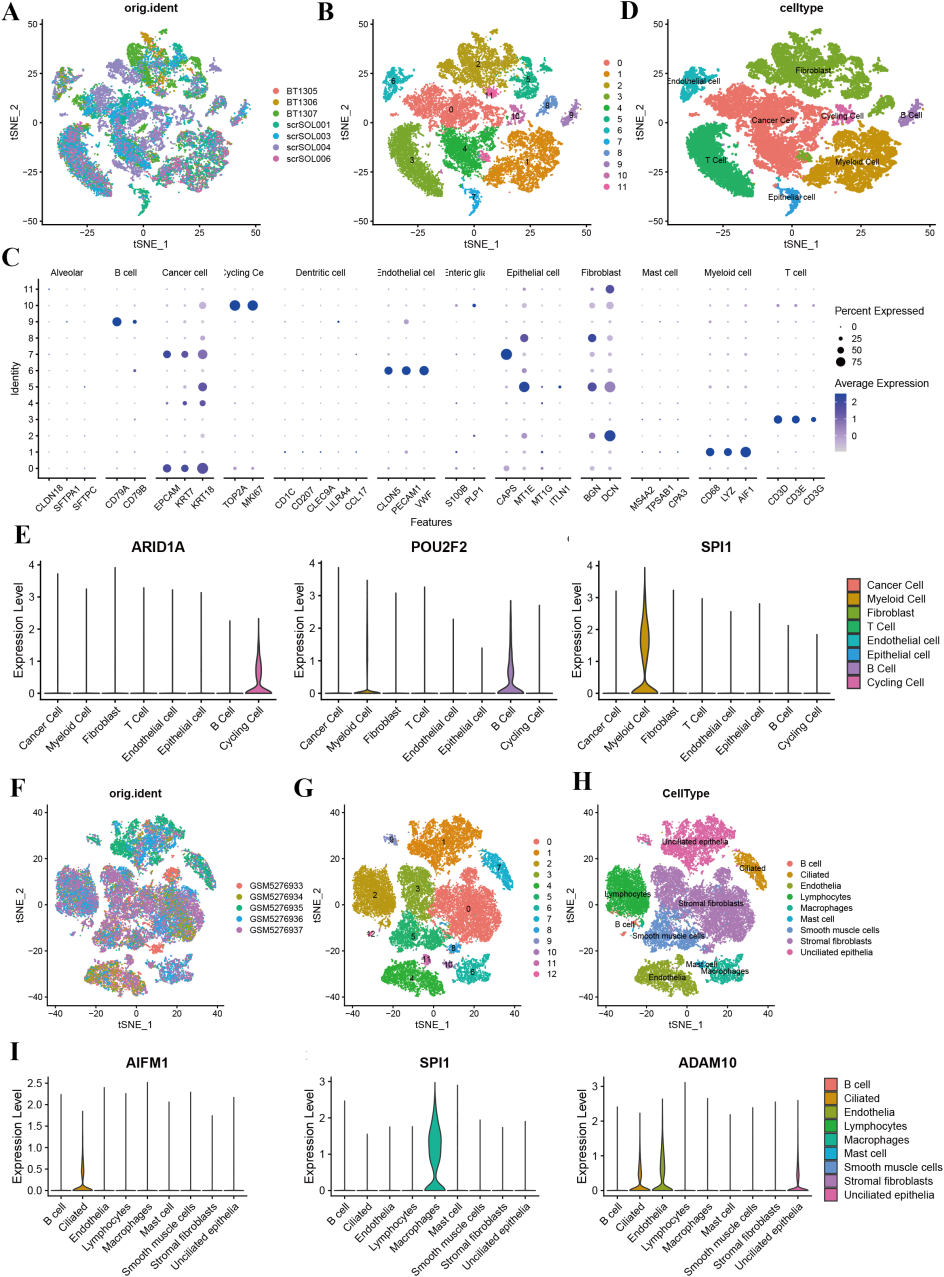
Single-cell sequencing analysis of ovarian cancer and endometrial cancer. **(A)** t-SNE plot showing cell distribution based on sample origin, with each color representing a distinct sample. **(B)** t-SNE plot depicting clustering results, with clusters labeled numerically (0–11). **(C)** Dot plot illustrating the expression levels of marker genes across various cell types. Dot size indicates the percentage of cells expressing the gene, and color intensity represents the average expression level. **(D)** Annotation of 12 clusters into eight different cell types in ovarian cancer. **(E)** The violin plot shows the expression of ARID1A across various cell types, with the highest levels observed in cancer cells, fibroblasts, and epithelial cells. Similarly, the violin plot highlights the expression of POU2F2, which is predominantly found in cancer cells and myeloid cells. The violin plot demonstrates that SPI1 is highly enriched in myeloid cells. **(F)** t-SNE plot showing the distribution of cells from five UCEC samples, with each color representing a distinct sample. **(G)** t-SNE plot illustrating clustering of cells into 13 distinct groups (0–12). **(H)** Annotation of 13 clusters into eight different cell types in endometrial cancer. **(I)** Violin plots displaying the expression levels of key genes (AIFM1, SPI1, and ADAM10) across the identified cell types. AIFM1 is predominantly expressed in macrophage; SPI1 is highly expressed in macrophages; and ADAM10 shows the highest expression in lymphocytes.

Single-cell RNA sequencing analysis revealed the cellular heterogeneity and distribution characteristics of key genes across different cell types in breast cancer, cervical cancer, ovarian cancer, and endometrial cancer. In breast cancer, t-SNE plots showed significant overlap in the distribution of cells across samples from different patients, suggesting shared cellular characteristics between samples. Clustering analysis divided the cells into 14 clusters, which were annotated as cancer cell, fibroblasts, cycling cell, and other cell types. Key gene expression analysis revealed that C17orf89 was primarily expressed in cancer cell and B cell, FDX1 showed the highest expression in fibroblasts and myeloid cell, and HMGB2 was highly expressed in cancer cell and cycling cell, suggesting its role in the tumor microenvironment and cell proliferation (**Figures 7A–E**). Analysis of cervical cancer indicated a high degree of overlap in cell distribution between samples, with cells divided into six clusters annotated as cancer cell, lymphocytes, and macrophages, where cancer cell was predominant. APMAP was mainly expressed in lymphocytes and cancer cell, SPI1 was highly expressed in macrophages, and ADAM10 showed high expression in cancer cell and lymphocytes, indicating their potential roles in tumor immunity and microenvironment regulation (**Figures 7F–J**). In ovarian cancer, cells from different samples showed highly similar characteristics and were clustered into 12 groups annotated as cancer cells, fibroblasts, endothelial cell, and myeloid cell, among others. ARID1A was highly expressed in cancer cell and fibroblasts, POU2F2 was significantly expressed in cancer cell, and SPI1 was highly specific to myeloid cell, suggesting its involvement in immune regulation (**Figures 8A–E**). In endometrial cancer, consistent cell distribution was observed across samples, with cells grouped into 13 clusters and annotated as B cell, lymphocytes, macrophages, ciliated epithelial cell, and smooth muscle cells, among others. Gene expression analysis showed that AIFM1 was highly expressed in endothelial cells and macrophages, SPI1 was significantly expressed in macrophages and mast cell, and ADAM10 was most highly expressed in lymphocytes (**Figures 8F–I**). These results comprehensively revealed the cellular heterogeneity and functional characteristics of key genes in the tumor microenvironment of different cancer types, providing important insights for further research into related mechanisms and therapeutic target exploration.

### 3.9 The effect of HMGB2 knockdown on cell proliferation

To investigate the impact of HMGB2 knockdown on cancer cell proliferation, CCK-8 and EdU fluorescence staining assays were performed in eight cancer cell lines representing breast, cervical, ovarian, and endometrial cancers. The results demonstrated that HMGB2 knockdown significantly suppressed proliferation across all tested cell lines.

**Figure 9:**
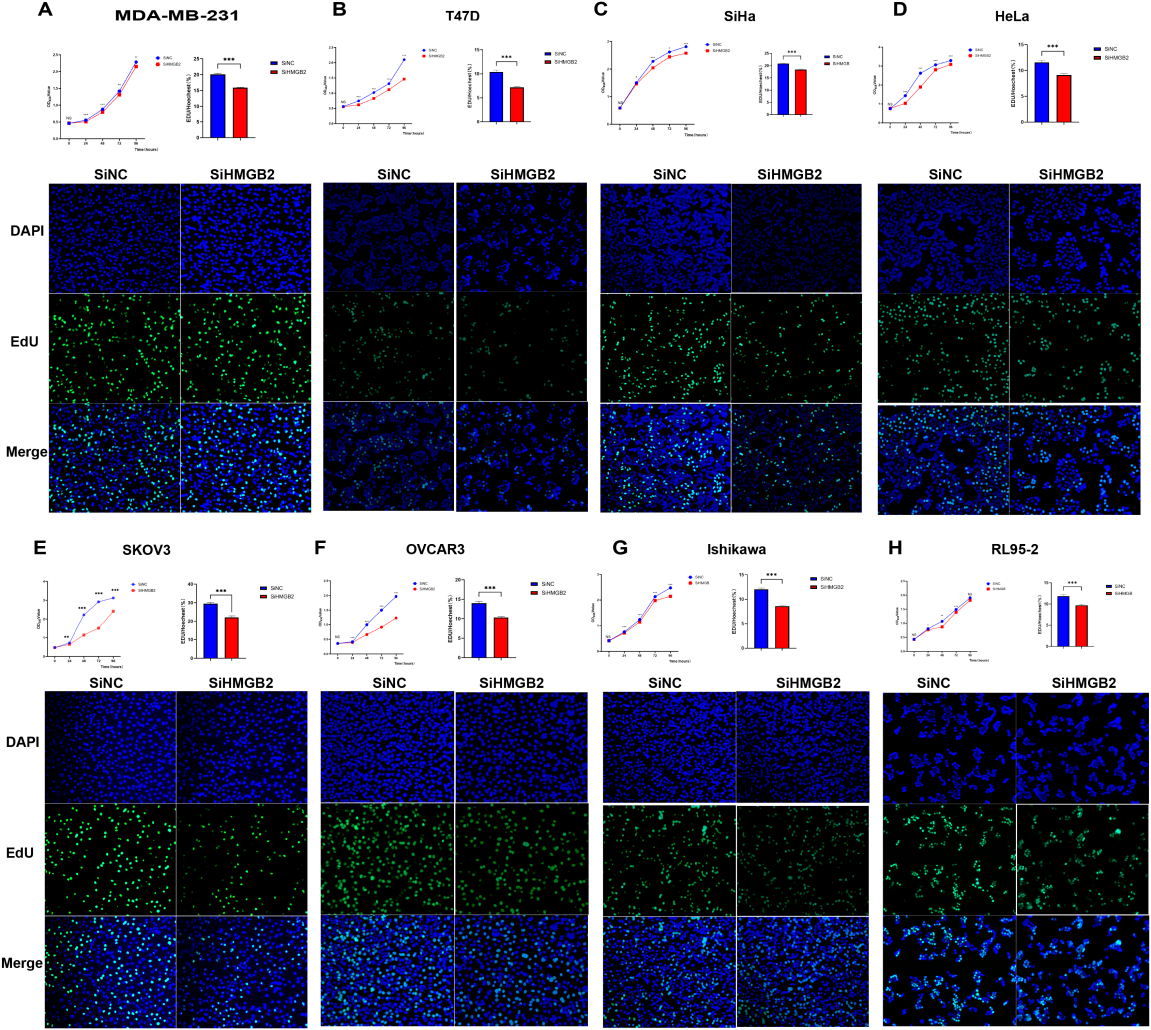
HMGB2 knockdown inhibits the proliferation of breast, cervical, ovarian, and endometrial cancer cell lines. **(A, B)** HMGB2 knockdown significantly reduced proliferation as indicated by decreased OD450 values in CCK-8 assays over time (MDA-MB-231: 24h, t = 8.090, P < 0.001; 48h, t = 10.94, P < 0.001; 72h, t = 4.468, P = 0.002; 96h, t = 3.840, P = 0.002; T47D: 24h, t = 21.43, P < 0.001; 48h, t = 20.61, P < 0.001; 72h, t = 9.565, P < 0.001; 96h, t = 19.66, P < 0.001). Similarly, EdU staining showed significant reductions in the percentage of EdU-positive cells (MDA-MB-231: SiNC = 20.088% ± 1.432%, SiHMGB2 = 15.859% ± 0.637%, t = 11.13, P < 0.001; T47D: SiNC = 10.385% ± 1.829%, SiHMGB2 = 7.188% ± 0.794%, t = 7.349, P < 0.001). **(C, D)** Cervical cancer cell lines (SiHa and HeLa): HMGB2 knockdown led to significant reductions in proliferation, as shown by decreases in OD450 values (SiHa: 24h, t = 2.232, P = 0.04; 48h, t = 16.78, P < 0.001; 72h, t = 2.974, P = 0.01; 96h, t = 13.08, P < 0.001; HeLa: 24h, t = 8.242, P < 0.001; 48h, t = 40.16, P < 0.001; 72h, t = 12.90, P < 0.001; 96h, t = 10.33, P < 0.001). Consistently, EdU fluorescence staining revealed reduced percentages of EdU-positive cells (SiHa: SiNC = 20.844% ± 0.894%, SiHMGB2 = 18.396% ± 0.985%, t = 7.578, P < 0.001; HeLa: SiNC = 11.580% ± 1.701%, SiHMGB2 = 9.119% ± 1.094%, t = 5.304, P < 0.001). **(E, F)** Ovarian cancer cell lines (SKOV3 and OVCAR3): HMGB2 knockdown significantly inhibited proliferation, with reduced OD450 values across all time points (SKOV3: 24h, t = 3.426, P = 0.004; 48h, t = 30.06, P < 0.001; 72h, t = 66.66, P < 0.001; 96h, t = 15.89, P < 0.001; OVCAR3: 24h, t = 5.510, P < 0.001; 48h, t = 11.56, P < 0.001; 72h, t = 17.56, P < 0.001; 96h, t = 14.44, P < 0.001). EdU staining revealed similar reductions in EdU-positive cells (SKOV3: SiNC = 29.455% ± 2.816%, SiHMGB2 = 22.072% ± 3.303%, t = 7.217, P < 0.001; OVCAR3: SiNC = 13.989% ± 2.144%, SiHMGB2 = 10.312% ± 1.142%, t = 6.599, P < 0.001). **(G, H)** Endometrial cancer cell lines (Ishikawa and RL95-2): HMGB2 knockdown consistently impaired proliferation. OD450 values in CCK-8 assays were significantly reduced at all time points (Ishikawa: 24h, t = 4.435, P < 0.001; 48h, t = 7.248, P < 0.001; 72h, t = 6.172, P < 0.001; 96h, t = 11.21, P < 0.001; RL95-2: 24h, t = 2.292, P = 0.04; 48h, t = 3.674, P = 0.003; 72h, t = 5.039, P < 0.001). EdU staining further confirmed significant reductions in EdU-positive cells (Ishikawa: SiNC = 12.025% ± 1.104%, SiHMGB2 = 8.604% ± 0.557%, t = 11.41, P < 0.001; RL95-2: SiNC = 11.860% ± 1.734%, SiHMGB2 = 9.685% ± 1.055%, t = 4.287, P < 0.001). *P < 0.05, **P < 0.01, ***P < 0.001.

In breast cancer cells, HMGB2 knockdown markedly reduced proliferation in both MDA-MB-231 and T47D cell lines. The CCK-8 assay revealed significant decreases in OD450 values over time (MDA-MB-231: 24h, t = 8.090, P < 0.001; 48h, t = 10.94, P < 0.001; 72h, t = 4.468, P = 0.002; 96h, t = 3.840, P = 0.002; T47D: 24h, t = 21.43, P < 0.001; 48h, t = 20.61, P < 0.001; 72h, t = 9.565, P < 0.001; 96h, t = 19.66, P < 0.001) (**Figures 9A, B**). Similarly, the EdU fluorescence staining assay showed that HMGB2 knockdown significantly decreased the percentage of EdU-positive cells in both MDA-MB-231 (SiNC = 20.088% ± 1.432%, SiHMGB2 = 15.859% ± 0.637%, t = 11.13, P < 0.001) and T47D (SiNC = 10.385% ± 1.829%, SiHMGB2 = 7.188% ± 0.794%, t = 7.349, P < 0.001) cells (**Figures 9A, B**).

In cervical cancer cells, similar results were observed in SiHa and HeLa cell lines. The CCK-8 assay demonstrated significant reductions in OD450 values at all time points (SiHa: 24h, t = 2.232, P = 0.04; 48h, t = 16.78, P < 0.001; 72h, t = 2.974, P = 0.01; 96h, t = 13.08, P < 0.001; HeLa: 24h, t = 8.242, P < 0.001; 48h, t = 40.16, P < 0.001; 72h, t = 12.90, P < 0.001; 96h, t = 10.33, P < 0.001) (**Figures 9C, D**). Consistently, EdU fluorescence staining showed a significant decrease in EdU-positive cells in SiHa (SiNC = 20.844% ± 0.894%, SiHMGB2 = 18.396% ± 0.985%, t = 7.578, P < 0.001) and HeLa (SiNC = 11.580% ± 1.701%, SiHMGB2 = 9.119% ± 1.094%, t = 5.304, P < 0.001) cells (**Figures 9C, D**).

In ovarian cancer cell lines SKOV3 and OVCAR3, HMGB2 knockdown also led to a marked reduction in proliferation. The CCK-8 assay indicated significant declines in OD450 values at all time points (SKOV3: 24h, t = 3.426, P = 0.004; 48h, t = 30.06, P < 0.001; 72h, t = 66.66, P < 0.001; 96h, t = 15.89, P < 0.001; OVCAR3: 24h, t = 5.510, P < 0.001; 48h, t = 11.56, P < 0.001; 72h, t = 17.56, P < 0.001; 96h, t = 14.44, P < 0.001) (**Figures 9E, F**). The percentage of EdU-positive cells also decreased significantly in SKOV3 (SiNC = 29.455% ± 2.816%, SiHMGB2 = 22.072% ± 3.303%, t = 7.217, P < 0.001) and OVCAR3 (SiNC = 13.989% ± 2.144%, SiHMGB2 = 10.312% ± 1.142%, t = 6.599, P < 0.001) cells (**Figures 9E, F**).

In endometrial cancer cells, Ishikawa and RL95-2, HMGB2 knockdown caused a consistent decrease in proliferation. CCK-8 assays showed significant reductions in OD450 values across all time points (Ishikawa: 24h, t = 4.435, P < 0.001; 48h, t = 7.248, P < 0.001; 72h, t = 6.172, P < 0.001; 96h, t = 11.21, P < 0.001; RL95-2: 24h, t = 2.292, P = 0.04; 48h, t = 3.674, P = 0.003; 72h, t = 5.039, P < 0.001) (**Figures 9G, H**). EdU staining confirmed these results, with significant reductions in EdU-positive cells in Ishikawa (SiNC = 12.025% ± 1.104%, SiHMGB2 = 8.604% ± 0.557%, t = 11.41, P < 0.001) and RL95-2 (SiNC = 11.860% ± 1.734%, SiHMGB2 = 9.685% ± 1.055%, t = 4.287, P < 0.001) cells (**Figures 9G, H**).

Together, these findings demonstrate that HMGB2 knockdown significantly inhibits cancer cell proliferation across a range of female-specific cancers, highlighting its critical role in tumor growth and suggesting its potential as a therapeutic target.

### 3.10 The effect of HMGB2 knockdown on cell invasion

The impact of HMGB2 knockdown on the invasive abilities of eight cancer cell lines representing breast cancer, cervical cancer, ovarian cancer, and endometrial cancer was analyzed using the Transwell invasion assay.

**Figure 10:**
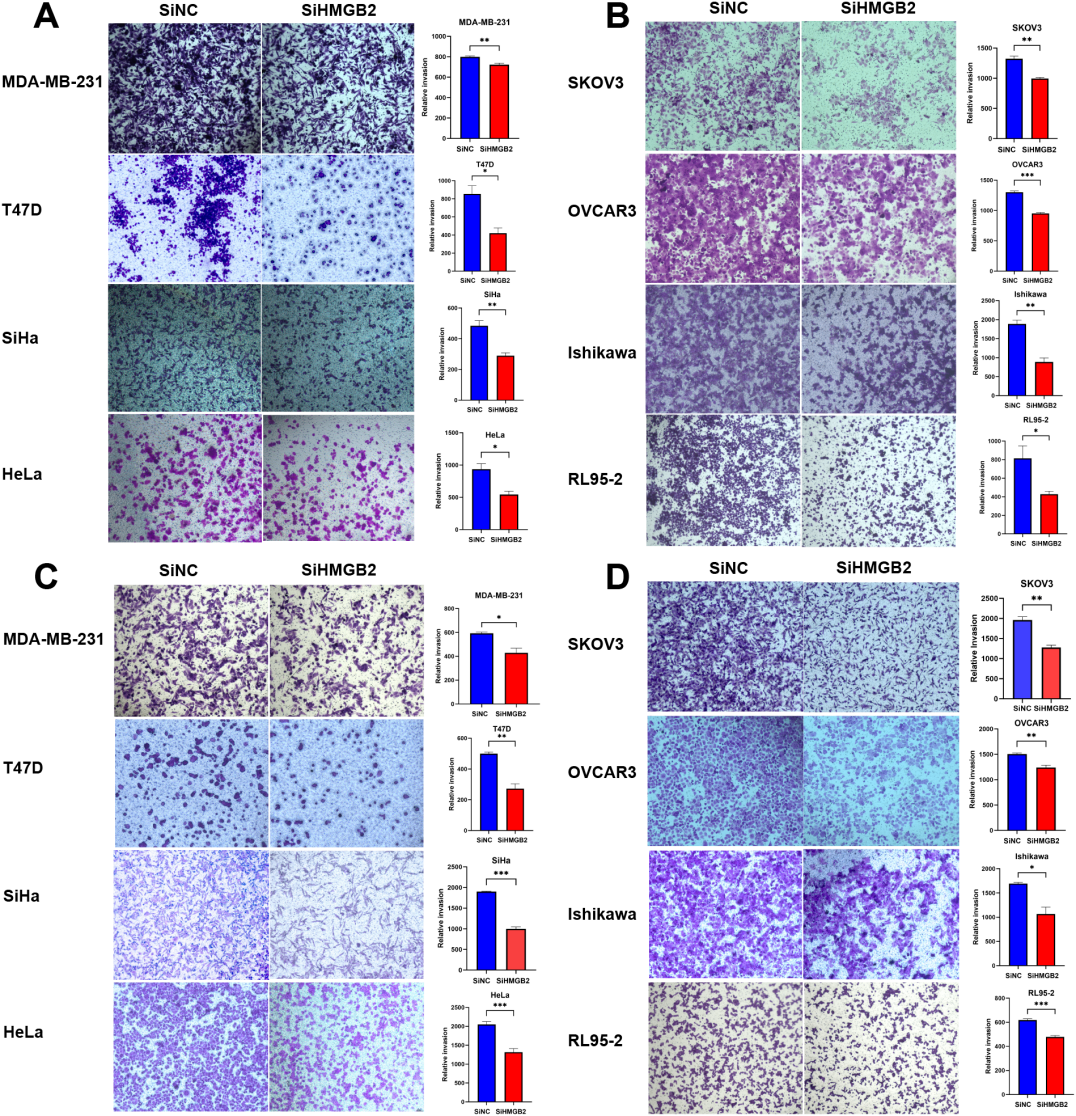
The impact of HMGB2 knockdown on the invasive and migratory abilities of breast, cervical, ovarian, and endometrial cancer cell lines. **(A, B)**. Invasion assay: Transwell assays were used to evaluate the invasive capacities of eight cancer cell lines after HMGB2 knockdown. Significant inhibition of invasion was observed across all cancer types. In breast cancer, invasive cell numbers decreased after 48 hours for MDA-MB-231 (t = 4.632, P = 0.010) and T47D (t = 3.993, P = 0.020). In cervical cancer, reductions were observed after 56 hours for SiHa (t = 5.132, P = 0.007) and 48 hours for HeLa (t = 3.972, P = 0.020). For ovarian cancer, SKOV3 showed reduced invasion after 56 hours (t = 7.093, P = 0.002), and OVCAR3 after 42 hours (t = 12.77, P < 0.001). In endometrial cancer, invasion was suppressed after 48 hours in Ishikawa (t = 6.668, P = 0.003) and after 56 hours in RL95-2 (t = 3.713, P = 0.020). **(C, D)**. Migration assay: Transwell assays demonstrated that HMGB2 knockdown significantly impaired migration across all cancer cell lines. In breast cancer, migration decreased after 24 hours for MDA-MB-231 (t = 4.052, P = 0.020) and 24 hours for T47D (t = 6.831, P = 0.002). In cervical cancer, migration was inhibited after 24 hours for both SiHa (t = 18.60, P < 0.001) and HeLa (t = 6.078, P < 0.001). For ovarian cancer, migration decreased after 24 hours in SKOV3 (t = 6.725, P = 0.003) and OVCAR3 (t = 5.213, P = 0.006). In endometrial cancer, Ishikawa and RL95–2 showed reduced migration after 24 hours (t = 4.339, P = 0.010 and t = 9.426, P < 0.001, respectively). *P < 0.05, **P < 0.01, and ***P < 0.001.

In breast cancer cell lines (MDA-MB-231 and T47D), HMGB2 knockdown significantly inhibited cell invasion. For MDA-MB-231 cells, the number of invasive cells was significantly reduced after 48 hours (t = 4.632, P = 0.010) (**Figure 10A**). Similarly, in T47D cells, HMGB2 knockdown resulted in a significant reduction in invasive ability at 48 hours (t = 3.993, P = 0.020) (**Figure 10A**).

In cervical cancer cell lines (SiHa and HeLa), HMGB2 knockdown significantly impaired cell invasion. For SiHa cells, the number of invasive cells was significantly reduced after 56 hours (t = 5.132, P = 0.007) (**Figure 10A**). In HeLa cells, a significant decrease in invasion was observed at 48 hours (t = 3.972, P = 0.020) (**Figure 10A**).

In ovarian cancer cell lines (SKOV3 and OVCAR3), HMGB2 knockdown markedly reduced cell invasion. In SKOV3 cells, the number of invasive cells decreased significantly after 56 hours (t = 7.093, P = 0.002) (**Figure 10B**). For OVCAR3 cells, the invasion ability was significantly inhibited after 48 hours (t = 12.77, P < 0.001) (**Figure 10B**).

In endometrial cancer cell lines (Ishikawa and RL95-2), HMGB2 knockdown significantly reduced cell invasion. For Ishikawa cells, the invasive ability was significantly impaired after 48 hours (t = 6.668, P = 0.003) (**Figure 10B**). Similarly, in RL95–2 cells, a reduction in invasive capacity was observed after 56 hours (t = 3.713, P = 0.020) (**Figure 10B**).

These findings demonstrate that HMGB2 knockdown significantly suppresses the invasive potential of cancer cells across multiple female cancer types.

### 3.11 The effect of HMGB2 knockdown on cell migration

The impact of HMGB2 knockdown on the migration abilities of eight cancer cell lines representing breast cancer, cervical cancer, ovarian cancer, and endometrial cancer was analyzed using the Transwell migration assay.

In breast cancer cell lines (MDA-MB-231 and T47D), HMGB2 knockdown significantly inhibited cell migration. For MDA-MB-231 cells, the migratory ability was significantly reduced after 24 hours (t = 4.052, P = 0.02) (**Figure 10C**). Similarly, in T47D cells, a significant decrease in migration was observed after 24 hours (t = 6.831, P = 0.002) (**Figure 10C**).

In cervical cancer cell lines (SiHa and HeLa), HMGB2 knockdown markedly impaired migration ability. In SiHa cells, the number of migrating cells was significantly reduced after 24 hours (t = 18.60, P < 0.001) (**Figure 10C**). Similarly, in HeLa cells, HMGB2 knockdown resulted in a significant reduction in migration at 24 hours (t = 6.078, P < 0.001) (**Figure 10C**).

In ovarian cancer cell lines (SKOV3 and OVCAR3), HMGB2 knockdown significantly suppressed migration. In SKOV3 cells, migratory ability was reduced after 24 hours (t = 6.725, P = 0.003) (**Figure 10D**). In OVCAR3 cells, a similar significant reduction in migration was observed at 24 hours (t = 5.213, P = 0.006) (**Figure 10D**).

In endometrial cancer cell lines (Ishikawa and RL95-2), HMGB2 knockdown significantly impaired migration. For Ishikawa cells, the migratory ability was reduced after 24 hours (t = 4.339, P = 0.01) (**Figure 10D**). In RL95–2 cells, the number of migrating cells was significantly reduced after 24 hours (t = 9.426, P < 0.001) (**Figure 10D**).

These findings indicate that HMGB2 knockdown significantly suppresses the migratory potential of cancer cells across multiple female cancer types.

### 3.12 Effect of HMGB2 knockdown in macrophages on tumor cell phagocytosis

To investigate the role of HMGB2 in macrophages on tumor cell phagocytosis, we performed co-culture assays using the murine macrophage cell line RAW264.7 and four human female-specific tumor cell lines: MDA-MB-231 (breast cancer), HeLa (cervical cancer), SKOV3 (ovarian cancer), and Ishikawa (endometrial cancer). HMGB2 expression was knocked down in RAW264.7 cells, and the phagocytic activity toward each tumor cell type was assessed using a fluorescence microscopy-based phagocytosis assay. In all models tested, HMGB2 knockdown in macrophages led to a significant reduction in phagocytic activity. Specifically, RAW264.7 cells with HMGB2 knockdown showed a marked decrease in their ability to phagocytose MDA-MB-231 cells (t = 12.89, P < 0.001), HeLa cells (t = 7.433, P < 0.001), SKOV3 cells (t = 16.20, P < 0.001), and Ishikawa cells (P < 0.001) (**Supplementary Figure 1**). These results indicate that HMGB2 plays a critical role in maintaining macrophage-mediated phagocytic function across various female-specific cancer types.

### 3.13 Effect of HMGB2 knockdown combined with palbociclib on tumor cell proliferation

To investigate the effect of HMGB2 knockdown on tumor cell proliferation in the presence of Palbociclib, we conducted CCK8 assays on multiple cancer cell lines (**Supplementary Figure 2**).

For MDA-MB-231 and T47D cells, HMGB2 was knocked down, and the cells were treated with Palbociclib. The results showed a significant reduction in cell proliferation at all time points (MDA-MB-231, 24h, t = 22.30, P < 0.001; 48h, t = 23.72, P < 0.001; 72h, t = 51.45, P < 0.001; 96h, t = 135.3, P < 0.001; T47D, 24h, t = 7.096, P < 0.001; 48h, t = 12.67, P < 0.001; 72h, t = 16.90, P < 0.001; 96h, t = 18.43, P < 0.001) (**Supplementary Figures 2A, B**).

For HeLa and SiHa cells, following HMGB2 knockdown and Palbociclib treatment, significant inhibition of cell proliferation was observed (HeLa, 24h, t = 2.287, P = 0.05; 48h, t = 16.57, P < 0.001; 72h, P < 0.001; 96h, t = 18.46, P < 0.001;SiHa, 24h, t = 3.741, P = 0.002; 48h, t = 0.5503, P = 0.59; 72h, t = 8.826, P < 0.001; 96h, t = 23.26, P < 0.001) (**Supplementary Figures 2C, D**).

For SKOV3 and OVCAR3 cells, HMGB2 knockdown followed by Palbociclib treatment resulted in a marked decrease in cell proliferation (SKOV3, 24h, t = 8.311, P < 0.001; 48h, t = 30.15, P < 0.001; 72h, P < 0.001; 96h, t = 32.26, P < 0.001; OVCAR3, 24h, t = 14.89, P < 0.001; 48h, t = 5.809, P < 0.001; 72h, t = 42.66, P < 0.001; 96h, t = 55.20, P < 0.001) (**Supplementary Figures 2E, R**).

For Ishikawa and RL95–2 cells, HMGB2 knockdown followed by Palbociclib treatment also significantly reduced cell proliferation (Ishikawa: 24h, t = 24.14, P < 0.001; 48h, t = 61.09, P < 0.001; 72h, t = 106.8, P < 0.001; 96h, t = 106.1, P < 0.001; RL95-2: 24h, t = 10.67, P < 0.001; 48h, t = 10.64, P < 0.001; 72h, t = 24.28, P < 0.001) (**Supplementary Figures 2G, H**).

These results suggest that HMGB2 knockdown, when combined with Palbociclib, significantly inhibits the proliferation of a variety of cancer cell lines, demonstrating the potential therapeutic benefit of targeting HMGB2 in combination with cell cycle inhibitors.

To explore the impact of HMGB2 knockdown combined with Palbociclib treatment on tumor cell proliferation, we conducted EdU fluorescence staining assays on multiple cancer cell lines. Following HMGB2 knockdown, cells were treated with Palbociclib, and proliferation was measured by the percentage of EdU-positive cells (**Supplementary Figure 2**).

In breast cancer cell lines, MDA-MB-231 and T47D, HMGB2 knockdown significantly decreased the proportion of EdU-positive cells after Palbociclib treatment. Specifically, the percentage of EdU-positive cells in the MDA-MB-231 cell line decreased from 12.54% ± 0.32% (SiHMGB2) to 8.45% ± 0.10% (SiHMGB2 + Palbociclib) (t = 12.12, P < 0.001). Similarly, for T47D cells, the percentage decreased from 11.96% ± 0.21% (SiHMGB2) to 7.70% ± 0.16% (SiHMGB2 + Palbociclib) (t = 16.25, P < 0.001) (**Supplementary Figures 2A, B**).

In cervical cancer cells, SiHa and HeLa, HMGB2 knockdown followed by Palbociclib treatment also led to a significant reduction in the percentage of EdU-positive cells. For SiHa cells, the percentage decreased from 16.27% ± 0.31% (SiHMGB2) to 11.28% ± 0.31% (SiHMGB2 + Palbociclib) (t = 11.34, P < 0.001), while in HeLa cells, it decreased from 14.11% ± 0.31% (SiHMGB2) to 5.07% ± 0.18% (SiHMGB2 + Palbociclib) (t = 25.44, P < 0.001) (**Supplementary Figures 2C, D**).

In ovarian cancer models, SKOV3 and OVCAR3 cells, HMGB2 knockdown followed by Palbociclib treatment resulted in significant inhibition of cell proliferation. In SKOV3 cells, the percentage of EdU-positive cells dropped from 10.57% ± 0.23% (SiHMGB2) to 7.22% ± 0.13% (SiHMGB2 + Palbociclib) (t = 12.85, P < 0.001), while in OVCAR3 cells, it decreased from 11.99% ± 0.27% (SiHMGB2) to 7.93% ± 0.09% (SiHMGB2 + Palbociclib) (t = 14.24, P < 0.001) (**Supplementary Figures 2E, F**).

Endometrial cancer cells (Ishikawa and RL95-2) also showed a significant reduction in proliferation after HMGB2 knockdown and Palbociclib treatment. In Ishikawa cells, the percentage of EdU-positive cells decreased from 10.55% ± 0.16% (SiHMGB2) to 6.80% ± 0.15% (SiHMGB2 + Palbociclib) (t = 17.15, P < 0.001), and in RL95–2 cells, it decreased from 14.38% ± 0.63% (SiHMGB2) to 9.75% ± 0.30% (SiHMGB2 + Palbociclib) (t = 6.61, P < 0.001) (**Supplementary Figures 2G, H**).

## 4 Discussion

Female cancers, including breast cancer, cervical cancer, ovarian cancer, and endometrial cancer, are among the most common malignancies affecting women worldwide, posing a serious threat to women’s health and lives. In recent years, increasing evidence has demonstrated that phagocytosis plays a critical role within the tumor immune microenvironment, influencing tumor initiation and progression (18).

The present study aimed to explore the association between phagocytosis regulators and the clinical features of female-specific cancers, including breast, cervical, ovarian, and endometrial cancers. Through a systematic analysis of multiple datasets, including TCGA and GEO, this study examined the expression patterns, genomic variations, and epigenetic modifications of phagocytosis regulators, alongside functional validations to elucidate their role in tumor progression.

Our analysis revealed significant differential expression of phagocytosis regulators across the four cancer types. Notably, CD47 exhibited high expression in all cancers, consistent with its role in immune evasion by inhibiting macrophage-mediated phagocytosis. In contrast, FOXO1 was consistently downregulated, highlighting its potential as a tumor suppressor. Further investigation of genomic variations demonstrated high-frequency SNVs and CNVs in key regulators such as PTEN, ARID1A, and UBR4, particularly in endometrial cancer, where PTEN and ARID1A mutations were significantly enriched. Copy number alterations in genes such as PDCD10 and NDUFB9 (amplifications) and NDUFS7 and ZBTB7A (deletions) were also identified, particularly in ovarian cancer, suggesting their contributions to genomic instability. Epigenetic regulation of phagocytosis regulators was another crucial finding, with DNA methylation significantly influencing gene expression. For instance, HMGB1, HMGB2, and MS4A1 were hypomethylated and highly expressed, whereas hypermethylation of genes such as LDB1, NDUFS2, and POU2F2 led to their reduced expression, reinforcing the impact of DNA methylation on gene dysregulation in cancer.

The functional role of HMGB2, a critical phagocytosis regulator, was validated through *in vitro* experiments. HMGB2 knockdown significantly suppressed cancer cell proliferation, migration, and invasion across breast, cervical, ovarian, and endometrial cancer cell lines. CCK-8 and EdU assays revealed that HMGB2 inhibition reduced cell proliferation, while Transwell assays demonstrated impaired migratory and invasive capacities. These results underscore HMGB2’s essential role in promoting cancer progression, potentially by regulating cell cycle progression, metabolic reprogramming, and immune evasion mechanisms.

In this study, we further investigated the potential therapeutic implications of HMGB2 inhibition in combination with Palbociclib, a small-molecule drug that targets the cell cycle. Our results demonstrated that the combination of HMGB2 knockdown and Palbociclib treatment significantly inhibited cell proliferation, as evidenced by the CCK8 and EdU assays. Compared to the control group, where only HMGB2 was knocked down, the experimental group, which received Palbociclib treatment, exhibited a marked reduction in cell proliferation. This suggests that targeting HMGB2, in combination with cell cycle inhibitors such as Palbociclib, can effectively suppress tumor cell growth.

### 4.1 Expression of phagocytosis regulators and female tumorigenesis and development

CD47, a transmembrane protein, transmits a “don’t eat me” signal by binding to the macrophage surface receptor SIRPα, thereby inhibiting macrophage phagocytosis and facilitating tumor cell immune evasion (19).

In breast cancer, CD47 expression is upregulated by hypoxia-inducible factor 1 (HIF-1), particularly under hypoxic conditions. CD47 not only promotes immune evasion but also sustains the survival of cancer stem cells (20). Moreover, the co-expression of CD47 with CD68, a macrophage marker, is significantly associated with the high invasiveness and poor prognosis of hormone receptor-negative breast cancer (21). In ovarian cancer, high CD47 expression is similarly correlated with poor clinical outcomes, primarily by suppressing macrophage phagocytic activity and promoting tumor immune evasion (22). Notably, therapeutic blockade of CD47 has shown promising potential in enhancing macrophage-mediated phagocytosis of tumor cells, thereby inhibiting ovarian cancer progression (23). In endometrial cancer, CD47 overexpression further promotes immune evasion in tumor cells. Anti-CD47 therapies have been demonstrated to significantly enhance macrophage phagocytosis and suppress tumor growth, highlighting its potential as a promising therapeutic target (24).

### 4.2 The impact of genomic variations (SNVs and CNVs) on the expression of phagocytosis regulators

This study revealed that single-nucleotide variants (SNVs) and copy number variations (CNVs) significantly influence the expression of phagocytosis regulators, further impacting the progression and prognosis of female-specific cancers. For instance, copy number variations affect the expression of key protein kinase genes, such as MERTK and MET, which have been shown to correlate with survival outcomes in endometrial cancer and renal cancer (25). CNVs may also alter the expression of RNA methylation regulators, subsequently influencing the levels of immune cell infiltration and driving tumor progression (26). Furthermore, in this study, SNVs in phagocytosis regulators such as PTEN and ARID1A exhibited a significantly higher mutation frequency in endometrial cancer compared to other cancer types. This finding suggests that these genes may play essential tumor suppressive roles in female-specific cancers.

### 4.3 The impact of DNA methylation on the expression of phagocytosis regulators

DNA methylation, as a critical component of epigenetic modifications, plays a key regulatory role in the expression of phagocytosis regulators. DNA methylation inhibitors (DNMTis) have been shown to activate endogenous retroviral (ERV) elements, thereby enhancing interferon signaling pathways, promoting tumor cell apoptosis, and activating immune pathways (27). In addition, the expression of CD47 is regulated by specific microRNAs, such as miR-133a, which can suppress CD47 expression and enhance macrophage-mediated phagocytosis (28). FOXO1, as a critical tumor suppressor, antagonizes the function of the epigenetic repressor EZH2. EZH2 silences tumor suppressor genes, such as CDKN2A, through methylation. Downregulation of FOXO1 may promote methylation-mediated gene silencing, thereby accelerating tumor progression (29). Pan-cancer studies have demonstrated that DNA methylation frequently suppresses tumor suppressor gene expression. For example, upregulation of DNA methyltransferase DNMT1 can induce hypermethylation of key tumor suppressor genes, such as p16^INK4a, thereby promoting tumor progression (30, 31). Additionally, DNA methylation can regulate the expression of specific elements, such as ERVs, which may play crucial roles in both immune evasion and tumor immune activation (27).

### 4.4 The association between phagocytosis regulators and key biological pathways

In this study, key pathways such as the PI3K/AKT and HIPPO pathways were found to be closely associated with phagocytosis regulators. These pathways collectively participate in tumor proliferation, metastasis, and immune evasion mechanisms:

Members of the FOXO transcription factor family, including FOXO1, are inactivated through phosphorylation upon activation of the PI3K/AKT pathway. This inactivation limits FOXO1’s regulation of tumor suppressor genes, thereby promoting tumor progression (32). HMGB1 promotes the activation of the PI3K/AKT signaling pathway, leading to the upregulation of HIF-1α, which in turn enhances VEGF expression, inducing tumor angiogenesis and cell migration. This effect is particularly significant in breast cancer (33). CD47 mediates immune evasion by inhibiting macrophage phagocytosis. Notably, PI3K/AKT inhibitors can alleviate CD47-mediated immune escape, thereby enhancing macrophage phagocytic activity against tumor cells (24–36).

### 4.5 Clinical functional validation and mechanistic exploration of HMGB2

Our study provides compelling evidence that HMGB2 plays a critical role in regulating the proliferative, migratory, and invasive capacities of cancer cells in female-specific cancers, including breast, cervical, ovarian, and endometrial cancers. HMGB2 knockdown significantly inhibited these malignant phenotypes, suggesting its potential as a therapeutic target and a key driver of tumor progression.

HMGB2 knockdown reduced cell proliferation in all tested cancer types, as demonstrated by CCK-8 and EdU assays. Notable reductions in proliferation were observed in breast (MDA-MB-231, T47D), cervical (SiHa, HeLa), ovarian (SKOV3, OVCAR3), and endometrial (Ishikawa, RL95-2) cancer cells, indicating disrupted DNA synthesis and cell cycle progression.

These findings indicate that HMGB2 promotes cancer cell proliferation likely by modulating cell cycle progression and DNA replication. Given its role in regulating chromatin dynamics and transcriptional activity, HMGB2 may influence the expression of genes critical for cell cycle progression, such as cyclins and CDKs.

HMGB2 knockdown significantly inhibited the migration and invasion of cancer cells, as shown by Transwell assays across multiple female-specific cancer types. In breast (MDA-MB-231, T47D), cervical (SiHa, HeLa), ovarian (SKOV3, OVCAR3), and endometrial (Ishikawa, RL95-2) cancers, HMGB2 knockdown consistently reduced migratory and invasive capacities. The effects were particularly pronounced in highly metastatic cell lines like MDA-MB-231 and SKOV3, underscoring HMGB2’s role in promoting tumor aggressiveness.

These findings suggest that HMGB2 plays a critical role in promoting migration and invasion, underscoring its potential as a promising therapeutic target for inhibiting metastasis.

### 4.6 HMGB2 clinical functional validation and mechanistic exploration

HMGB2, a member of the high-mobility group box (HMG-box) family, has emerged as a critical regulator of tumor progression through its involvement in cell cycle regulation, metabolic reprogramming, and immune evasion mechanisms. Our results demonstrated that HMGB2 knockdown significantly inhibits cancer cell proliferation, migration, and invasion across multiple female-specific cancers. These findings align with previous studies and provide further evidence that HMGB2 exerts its oncogenic roles by modulating various signaling pathways.

HMGB2 plays a crucial role in regulating cell cycle progression, particularly at the G1/S and G2/M transitions, which are essential checkpoints in cancer proliferation. High HMGB2 expression promotes G2/M progression by impairing DNA damage repair and bypassing checkpoints through p53 suppression and reduced p21 activation (37, 38). It regulates cyclin A2 and cyclin B expression, key drivers of G2/M transition, and its knockdown disrupts cyclin-CDK activity, inhibiting tumor cell proliferation (39). HMGB2 also interacts with the WEE1/CDK1 pathway, enhancing sensitivity to WEE1 inhibition and inducing mitotic catastrophe (40). Additionally, HMGB2 supports the RB/E2F pathway to sustain G1/S progression by driving transcription of E2F-dependent genes (41). Collectively, HMGB2 facilitates uncontrolled tumor growth by maintaining cyclin-CDK activity and evading checkpoint mechanisms.

HMGB2 plays a key role in tumor metabolic reprogramming by promoting aerobic glycolysis (Warburg effect) over oxidative phosphorylation. It enhances the expression of LDHB (lactate dehydrogenase B) while suppressing FBP1(fructose-1,6-bisphosphatase), driving glycolysis to support energy production and biosynthetic needs for rapid proliferation. HMGB2 knockdown disrupts this metabolic shift, reducing lactate production and tumor growth (42). Additionally, HMGB2 modulates AMPK signaling to maintain energy balance under metabolic stress and is linked to mitochondrial function, enabling tumor adaptation to hypoxia (43). Though evidence on HMGB2’s specific role in PI3K/AKT/mTOR signaling is limited, similar effects to HMGB1 are likely, enhancing glycolysis via mTOR activation (44). Targeting HMGB2, combined with glycolysis inhibitors like 2-DG(2-deoxyglucose), has shown promise in reducing tumor progression and inducing energy stress in cancer cells (45).

HMGB2 plays a critical role in immune escape by modulating the tumor immune microenvironment (TME) and enhancing immune checkpoint pathways. It promotes PD-L1 expression, likely via NF-κB signaling, similar to HMGB1, facilitating T cell exhaustion. Combining HMGB2 inhibition with anti-PD-L1 therapies has been shown to restore T cell function and enhance anti-tumor immunity (46, 47). HMGB2 may also regulate CD47, suppressing macrophage phagocytosis, and dual targeting of CD47 and PD-L1 improves both phagocytosis and T cell-mediated clearance (47). Furthermore, HMGB2 drives the recruitment of tumor-associated macrophages (TAMs), MDSCs, and Tregs, promoting an immunosuppressive TME via RAGE and TLR4 signaling (48, 49). It also suppresses dendritic cell (DC) function, impairing T cell priming and creating a tolerogenic environment. These findings highlight HMGB2 as a promising immunotherapeutic target.

HMGB2, a key regulator of cell cycle progression, metabolic reprogramming, and immune evasion, represents a promising therapeutic target. Its knockdown enhances sensitivity to cell cycle inhibitors like Palbociclib (50), while combining HMGB2 inhibition with glycolysis inhibitors reduces tumor viability (45). Additionally, HMGB2 inhibition may improve immune checkpoint therapies by restoring T cell function and reducing immunosuppressive cells in the TME (51). Its association with poor prognosis and chemoresistance further underscores its potential as a biomarker and therapeutic target (52, 53).

HMGB2 drives tumor progression by regulating cell cycle checkpoints, metabolic pathways, and immune evasion mechanisms. Its knockdown inhibits cancer cell proliferation, disrupts glycolytic reprogramming, and restores anti-tumor immunity. Targeting HMGB2, either alone or in combination with cell cycle inhibitors, metabolic inhibitors, or immunotherapy, represents a promising strategy for improving outcomes in female-specific cancers.

### 4.7 Immune microenvironment and drug sensitivity

The immune microenvironment plays a pivotal role in tumor progression and therapeutic response, with phagocytosis regulators emerging as key modulators. Our analysis revealed a positive correlation between phagocytosis scores and immune cell infiltration in female-specific cancers, suggesting their predictive value for immunotherapy efficacy. Phagocytosis regulators, such as CD47 and HMGB1, significantly influence immune evasion, immune cell activity, and drug sensitivity.

CD47, a “don’t eat me” signal, inhibits macrophage-mediated phagocytosis by binding SIRPα, contributing to immune escape and poor prognosis (54). CD47 blockade enhances macrophage activity, dendritic cell cross-priming, and T cell activation, boosting antitumor immunity (55). Similarly, HMGB1, a damage-associated molecular pattern (DAMP) molecule, recruits macrophages and dendritic cells to the tumor microenvironment, linking innate and adaptive immunity (56). Furthermore, phagocytosis regulators modulate immune checkpoints like PD-L1 and synergize with inhibitors such as anti-PD-1/PD-L1 to amplify T cell responses and tumor clearance (57, 58).

Phagocytosis regulators also impact drug sensitivity. CD47 overexpression promotes chemoresistance by suppressing phagocytosis and apoptosis, while targeting CD47 with miR-155 restores sensitivity to agents like bortezomib (59). HMGB1 interacts with DNA damage repair and cell cycle regulators, suggesting potential crosstalk with pathways targeted by drugs like Palbociclib (60, 61). Targeting phagocytosis regulators in combination with glycolysis inhibitors or immune checkpoint therapies offers significant therapeutic potential.

Emerging clinical trials targeting the CD47-SIRPα axis have shown enhanced macrophage-mediated phagocytosis and tumor clearance, particularly when combined with checkpoint inhibitors or chemotherapy (62, 63).

We cultured HMGB2 knockdown cells with Palbociclib and observed a significant reduction in cell proliferation. These findings provide further evidence of the critical role HMGB2 plays in regulating cell cycle progression and highlight its potential as a therapeutic target. The observed synergistic effect of Palbociclib with HMGB2 inhibition suggests that combining HMGB2 modulation with cell cycle inhibitors could provide a promising strategy to enhance the efficacy of current cancer therapies, particularly in female-specific cancers where HMGB2 is implicated in tumor progression. These results warrant further exploration of combination therapies targeting both cell cycle regulation and immune evasion pathways for improved clinical outcomes.

In this study, we investigated the role of HMGB2 in macrophage-mediated phagocytosis across several cancer types. Our results provide compelling evidence that HMGB2 plays a crucial role in maintaining macrophage phagocytic activity, particularly in the context of female-specific cancers.

Firstly, using fluorescence microscopy-based phagocytosis assays, we demonstrated that HMGB2 knockdown in macrophages (RAW264.7 cells) led to a significant reduction in the phagocytic ability of these macrophages toward tumor cells from various cancer types, including breast cancer (MDA-MB-231), cervical cancer (HeLa), ovarian cancer (SKOV3), and endometrial cancer (Ishikawa). These findings indicate that HMGB2 is essential for macrophages to effectively phagocytose tumor cells, and its absence impairs this critical immune function. The observed reduction in phagocytosis highlights HMGB2’s potential as a modulator of macrophage-mediated immune responses in cancer.

Additionally, we explored the therapeutic potential of targeting HMGB2 by combining HMGB2 knockdown with the cell cycle inhibitor Palbociclib. *In vitro* assays revealed a significant decrease in tumor cell proliferation across multiple cancer cell lines, including MDA-MB-231 (breast cancer), HeLa (cervical cancer), SKOV3 (ovarian cancer), and Ishikawa (endometrial cancer), following HMGB2 knockdown and Palbociclib treatment. These results suggest that HMGB2 knockdown, in combination with Palbociclib, effectively reduces cell proliferation in a variety of cancer models, underscoring the therapeutic potential of targeting HMGB2 in conjunction with cell cycle inhibitors.

The significant reduction in both phagocytic activity and cell proliferation upon HMGB2 knockdown suggests that HMGB2 could serve as a promising therapeutic target, not only for modulating immune responses but also for enhancing the efficacy of cancer treatments. The combined approach of targeting HMGB2 and cell cycle regulators like Palbociclib may offer a new avenue for treating cancers, particularly those where HMGB2 is implicated in tumor progression and immune evasion.

This study has limitations, including reliance on publicly available datasets and the lack of *in vivo* validation. Future research should address these by using animal models to explore the role of HMGB2 in tumor progression and metastasis. *In vivo* studies will provide insights into how HMGB2 influences tumor growth and spread. Additionally, clinical investigations are necessary to assess HMGB2 and other phagocytosis regulators as biomarkers for prognosis and therapeutic targeting. Future studies should focus on collecting clinical samples to evaluate their expression and link to patient outcomes. Investigating their role in enhancing immunotherapy efficacy is also important. Longitudinal studies and clinical trials could help validate these biomarkers for early detection and therapeutic targeting, while testing combination therapies could offer new strategies to improve patient outcomes.

## 5 Conclusion

This study offers important insights into the molecular mechanisms driving female-specific cancers by elucidating the pivotal role of phagocytosis regulators, particularly HMGB2, in tumor progression. These findings provide valuable evidence for advancing our understanding of the tumor microenvironment and highlight potential biomarkers and therapeutic strategies to enhance clinical outcomes in female-specific cancers.

## Data Availability

The original contributions presented in the study are included in the article/**Supplementary Material**. Further inquiries can be directed to the corresponding author.

## Supplementary material

Supplementary Figure 1 Phagocytic activity of RAW264.7 macrophages towards different tumor cell types following HMGB2 knockdown. A. RAW264.7 cells with HMGB2 knockdown (siHMGB2) exhibited reduced phagocytic activity compared to the control (siNC) in the HeLa cell. B. RAW264.7 cells with HMGB2 knockdown (siHMGB2) exhibited reduced phagocytic activity compared to the control (siNC) in the MDA-MB-231 cell. C. RAW264.7 cells with HMGB2 knockdown (siHMGB2) exhibited reduced phagocytic activity compared to the control (siNC) in the Ishikawa cell. D. RAW264.7 cells with HMGB2 knockdown (siHMGB2) exhibited reduced phagocytic activity compared to the control (siNC) in the SKOV3 cell. ***P < 0.001.

Supplementary Figure 2 Effect of HMGB2 knockdown and Palbociclib treatment on tumor cell proliferation, as assessed by EdU fluorescence staining. **(A)** Knockdown of HMGB2 in MDA-MB-231 cells followed by Palbociclib treatment significantly suppressed cell proliferation, as evidenced by CCK8 and EdU assays. **(B)** Knockdown of HMGB2 in T-47D cells followed by Palbociclib treatment significantly suppressed cell proliferation, as evidenced by CCK8 and EdU assays. **(C)** Knockdown of HMGB2 in HeLa cells followed by Palbociclib treatment significantly suppressed cell proliferation, as evidenced by CCK8 and EdU assays. **(D)** Knockdown of HMGB2 in SiHa cells followed by Palbociclib treatment significantly suppressed cell proliferation, as evidenced by CCK8 and EdU assays. **(E)** Knockdown of HMGB2 in SK-OV-3 cells followed by Palbociclib treatment significantly suppressed cell proliferation, as evidenced by CCK8 and EdU assays. **(F)** Knockdown of HMGB2 in OVCAR3 cells followed by Palbociclib treatment significantly suppressed cell proliferation, as evidenced by CCK8 and EdU assays. **(G)** Knockdown of HMGB2 in Ishikawa cells followed by Palbociclib treatment significantly suppressed cell proliferation, as evidenced by CCK8 and EdU assays. **(H)** Knockdown of HMGB2 in RL95–2 cells followed by Palbociclib treatment significantly suppressed cell proliferation, as evidenced by CCK8 and EdU assays. NS>0.05,*P < 0.001, **P < 0.001, ***P < 0.001.

Supplementary Table 1 phagocytosis regulators and their promoter DNA methylation patterns.

Supplementary Table 3 The relationship between phagocytosis regulators and overall survival.

## References

[1] Sung H Ferlay J Siegel RL Laversanne M Soerjomataram I Jemal A. Global cancer statistics 2020: GLOBOCAN estimates of incidence and mortality worldwide for 36 cancers in 185 countries. CA Cancer J Clin. (2021) 71:209–49. doi: 10.3322/caac.21660 33538338

[2] Bray F Ferlay J Soerjomataram I Siegel RL Torre LA Jemal A. Global cancer statistics 2018: GLOBOCAN estimates of incidence and mortality worldwide for 36 cancers in 185 countries. CA Cancer J Clin. (2018) 68:394–424. doi: 10.3322/caac.21492 30207593

[3] Reid BM Permuth JB Sellers TA. Epidemiology of ovarian cancer: a review. Cancer Biol Med. (2017) 14:9–32. doi: 10.20892/j.issn.2095-3941.2016.0084 28443200 PMC5365187

[4] Lortet-Tieulent J Ferlay J Bray F Jemal A. International patterns and trends in endometrial cancer incidence, 1978-2013. J Natl Cancer Inst. (2018) 110:354–61. doi: 10.1093/jnci/djx214 29045681

[5] Fitzmaurice C Abate D Abbasi N Abbastabar H Abd-Allah F Abdel-Rahman O. Global, regional, and national cancer incidence, mortality, years of life lost, years lived with disability, and disability-adjusted life-years for 29 cancer groups, 1990 to 2017: A systematic analysis for the global burden of disease study. JAMA Oncol. (2019) 5:1749–68. doi: 10.1001/jamaoncol.2019.2996 PMC6777271 31560378

[6] Hoadley KA Yau C Hinoue T Wolf DM Lazar AJ Drill E. Cell-of-origin patterns dominate the molecular classification of 10,000 tumors from 33 types of cancer. Cell. (2018) 173:291–304.e6. doi: 10.1016/j.cell.2018.03.022 29625048 PMC5957518

[7] Liu C Xie J Lin B Tian W Wu Y Xin S. Pan-cancer single-cell and spatial-resolved profiling reveals the immunosuppressive role of APOE+ Macrophages in immune checkpoint inhibitor therapy. Adv Sci (Weinh). (2024) 11:e2401061. doi: 10.1002/advs.202401061 38569519 PMC11186051

[8] Weinstein JN Collisson EA Mills GB Shaw KR Ozenberger BA Ellrott K. The Cancer Genome Atlas Pan-Cancer analysis project. Nat Genet. (2013) 45:1113–20. doi: 10.1038/ng.2764 PMC3919969 24071849

[9] Xie J Deng X Xie Y Zhu H Liu P Deng W. Multi-omics analysis of disulfidptosis regulators and therapeutic potential reveals glycogen synthase 1 as a disulfidptosis triggering target for triple-negative breast cancer. MedComm (2020). (2024) 5:e502. doi: 10.1002/mco2.v5.3 38420162 PMC10901283

[10] Tian B Pang Y Gao Y Meng Q Xin L Sun C. A pan-cancer analysis of the oncogenic role of Golgi transport 1B in human tumors. J Transl Int Med. (2023) 11:433–48. doi: 10.2478/jtim-2023-0002 PMC10732491 38130634

[11] Anderson NM Simon MC. The tumor microenvironment. Curr Biol. (2020) 30:R921–r5. doi: 10.1016/j.cub.2020.06.081 PMC8194051 32810447

[12] Gordon SR Maute RL Dulken BW Hutter G George BM McCracken MN. PD-1 expression by tumour-associated macrophages inhibits phagocytosis and tumour immunity. Nature. (2017) 545:495–9. doi: 10.1038/nature22396 PMC5931375 28514441

[13] Chao MP Alizadeh AA Tang C Myklebust JH Varghese B Gill S. Anti-CD47 antibody synergizes with rituximab to promote phagocytosis and eradicate non-Hodgkin lymphoma. Cell. (2010) 142:699–713. doi: 10.1016/j.cell.2010.07.044 20813259 PMC2943345

[14] Luo X Shen Y Huang W Bao Y Mo J Yao L. Blocking CD47-SIRPα Signal axis as promising immunotherapy in ovarian cancer. Cancer Control. (2023) 30:10732748231159706. doi: 10.1177/10732748231159706 36826231 PMC9969460

[15] Jia X Yan B Tian X Liu Q Jin J Shi J. CD47/SIRPα pathway mediates cancer immune escape and immunotherapy. Int J Biol Sci. (2021) 17:3281–7. doi: 10.7150/ijbs.60782 PMC8416724 34512146

[16] Huang R Kang T Chen S. The role of tumor-associated macrophages in tumor immune evasion. J Cancer Res Clin Oncol. (2024) 150:238. doi: 10.1007/s00432-024-05777-4 38713256 PMC11076352

[17] Liu J Xavy S Mihardja S Chen S Sompalli K Feng D. Targeting macrophage checkpoint inhibitor SIRPα for anticancer therapy. JCI Insight. (2020) 5(12):e134728. doi: 10.1172/jci.insight.134728 32427583 PMC7406266

[18] Vasileva AV Gladkova MG Ashniev GA Osintseva ED Orlov AV Kravchuk EV. Super-enhancers and their parts: from prediction efforts to pathognomonic status. Int J Mol Sci. (2024) 25(6):3103. doi: 10.3390/ijms25063103 38542080 PMC10969950

[19] Wu F Pang H Li F Hua M Song C Tang J. Progress in cancer research on the regulator of phagocytosis CD47, which determines the fate of tumor cells (Review). Oncol Lett. (2024) 27:256. doi: 10.3892/ol.2024.14389 38646501 PMC11027102

[20] Zhang H Lu H Xiang L Bullen JW Zhang C Samanta D. HIF-1 regulates CD47 expression in breast cancer cells to promote evasion of phagocytosis and maintenance of cancer stem cells. Proc Natl Acad Sci U S A. (2015) 112:E6215–23. doi: 10.1073/pnas.1520032112 PMC4653179 26512116

[21] Yuan J He H Chen C Wu J Rao J Yan H. Combined high expression of CD47 and CD68 is a novel prognostic factor for breast cancer patients. Cancer Cell Int. (2019) 19:238. doi: 10.1186/s12935-019-0957-0 31528120 PMC6737685

[22] Liu R Wei H Gao P Yu H Wang K Fu Z. CD47 promotes ovarian cancer progression by inhibiting macrophage phagocytosis. Oncotarget. (2017) 8:39021–32. doi: 10.18632/oncotarget.16547 PMC5503592 28380460

[23] Wang CL Lin MJ Hsu CY Lin HY Tsai HP Long CY. CD47 promotes cell growth and motility in epithelial ovarian cancer. BioMed Pharmacother. (2019) 119:109105. doi: 10.1016/j.biopha.2019.109105 31493748

[24] Gu S Ni T Wang J Liu Y Fan Q Wang Y. CD47 blockade inhibits tumor progression through promoting phagocytosis of tumor cells by M2 polarized macrophages in endometrial cancer. J Immunol Res. (2018) 2018:6156757. doi: 10.1155/2018/6156757 30525058 PMC6247569

[25] Arumilli S Liu H. Protein kinases in phagocytosis: promising genetic biomarkers for cancer. bioRxiv. (2024) 2024.10.09.617495. doi: 10.1101/2024.10.09.617495

[26] Wei W Liu C Wang C Wang M Jiang W Zhou Y. Comprehensive pan-cancer analysis of N7-methylguanosine regulators: Expression features and potential implications in prognosis and immunotherapy. Front Genet. (2022) 13:1016797. doi: 10.3389/fgene.2022.1016797 36339001 PMC9633684

[27] Chiappinelli KB Strissel PL Desrichard A Li H Henke C Akman B. Inhibiting DNA Methylation Causes an Interferon Response in Cancer via dsRNA Including Endogenous Retroviruses. Cell. (2015) 162:974–86. doi: 0.1016/j.cell.2015.07.011 10.1016/j.cell.2015.07.011 PMC4556003 26317466

[28] Suzuki S Yokobori T Tanaka N Sakai M Sano A Inose T. CD47 expression regulated by the miR-133a tumor suppressor is a novel prognostic marker in esophageal squamous cell carcinoma. Oncol Rep. (2012) 28:465–72. doi: 10.3892/or.2012.1831 22641236

[29] Zhang Y Tong T. FOXA1 antagonizes EZH2-mediated CDKN2A repression in carcinogenesis. Biochem Biophys Res Commun. (2014) 453:172–8. doi: 10.1016/j.bbrc.2014.09.092 25264199

[30] Suzuki M Sunaga N Shames DS Toyooka S Gazdar AF Minna JD. RNA interference-mediated knockdown of DNA methyltransferase 1 leads to promoter demethylation and gene re-expression in human lung and breast cancer cells. Cancer Res. (2004) 64:3137–43. doi: 10.1158/0008-5472.CAN-03-3046 15126351

[31] Huang CY Ye ZH Huang MY Lu JJ. Regulation of CD47 expression in cancer cells. Transl Oncol. (2020) 13:100862. doi: 10.1016/j.tranon.2020.100862 32920329 PMC7494507

[32] Lin A Piao HL Zhuang L Sarbassov dos D Ma L Gan B. FoxO transcription factors promote AKT Ser473 phosphorylation and renal tumor growth in response to pharmacologic inhibition of the PI3K-AKT pathway. Cancer Res. (2014) 74:1682–93. doi: 10.1158/0008-5472.CAN-13-1729 PMC4087030 24448243

[33] He H Wang X Chen J Sun L Sun H Xie K. High-mobility group box 1 (HMGB1) promotes angiogenesis and tumor migration by regulating hypoxia-inducible factor 1 (HIF-1α) expression via the phosphatidylinositol 3-kinase (PI3K)/AKT signaling pathway in breast cancer cells. Med Sci Monit. (2019) 25:2352–60. doi: 10.12659/MSM.915690 PMC6454982 30930461

[34] Godfrey J Kang W Huang L Coma S Maute R P M. Macrophage activation by dual PI3K-δ/γ Inhibition enhances anti-CD47-mediated phagocytosis and prolongs survival in DLBCL. Blood. (2020) 136:40. doi: 10.1182/blood-2020-43304

[35] Du L Su Z Wang S Meng Y Xiao F Xu D. EGFR-induced and c-src-mediated CD47 phosphorylation inhibits TRIM21-dependent polyubiquitylation and degradation of CD47 to promote tumor immune evasion. Adv Sci (Weinh). (2023) 10:e2206380. doi: 10.1002/advs.202206380 37541303 PMC10520678

[36] Rausch M Tchaicha J Tibbitts T Henau O Sharma S Pink M. The PI3K-γ inhibitor, IPI-549, increases antitumor immunity by targeting tumor-associated myeloid cells and remodeling the immune-suppressive tumor microenvironment. Cancer Immunol Res. (2016) 4:B032–B. doi: 10.1158/2326-6066.IMM2016-B032

[37] Shimizu A Nishida J Ueoka Y Kato K Hachiya T Kuriaki Y. CyclinG contributes to G2/M arrest of cells in response to DNA damage. Biochem Biophys Res Commun. (1998) 242:529–33. doi: 10.1006/bbrc.1997.8004 9464250

[38] Zhang T Guan XW Gribben JG Liu FT Jia L. Blockade of HMGB1 signaling pathway by ethyl pyruvate inhibits tumor growth in diffuse large B-cell lymphoma. Cell Death Dis. (2019) 10:330. doi: 10.1038/s41419-019-1563-8 30988279 PMC6465275

[39] Li Y Peng L Seto E. Histone deacetylase 10 regulates the cell cycle G2/M phase transition via a novel let-7-HMGA2-cyclin A2 pathway. Mol Cell Biol. (2015) 35:3547–65. doi: 10.1128/MCB.00400-15 PMC4573710 26240284

[40] Schmidt M Rohe A Platzer C Najjar A Erdmann F Sippl W. Regulation of G2/M transition by inhibition of WEE1 and PKMYT1 kinases. Molecules. (2017) 22(12):2045. doi: 10.3390/molecules22122045 29168755 PMC6149964

[41] Sheldon LA. Inhibition of E2F1 activity and cell cycle progression by arsenic via retinoblastoma protein. Cell Cycle. (2017) 16:2058–72. doi: 10.1080/15384101.2017.1338221 PMC5731421 28880708

[42] Fu D Li J Wei J Zhang Z Luo Y Tan H. HMGB2 is associated with Malignancy and regulates Warburg effect by targeting LDHB and FBP1 in breast cancer. Cell Commun Signal. (2018) 16:8. doi: 10.1186/s12964-018-0219-0 29463261 PMC5819211

[43] Tang D Loze MT Zeh HJ Kang R. The redox protein HMGB1 regulates cell death and survival in cancer treatment. Autophagy. (2010) 6:1181–3. doi: 10.4161/auto.6.8.13367 20861675

[44] Yu L Lu M Jia D Ma J Ben-Jacob E Levine H. Modeling the genetic regulation of cancer metabolism: interplay between glycolysis and oxidative phosphorylation. Cancer Res. (2017) 77:1564–74. doi: 10.1158/0008-5472.CAN-16-2074 PMC5380541 28202516

[45] Cai X Ding H Liu Y Pan G Li Q Yang Z. Expression of HMGB2 indicates worse survival of patients and is required for the maintenance of Warburg effect in pancreatic cancer. Acta Biochim Biophys Sin (Shanghai). (2017) 49:119–27. doi: 10.1093/abbs/gmw124 28069585

[46] Simmons G. 47461 Regulation of the immune response in the tumor microenvironment of lung adenocarcinoma. J Clin Trans Sci. (2021) 5:14–. doi: 10.1017/cts.2021.438

[47] Chen SH Dominik PK Stanfield J Ding S Yang W Kurd N. Dual checkpoint blockade of CD47 and PD-L1 using an affinity-tuned bispecific antibody maximizes antitumor immunity. J Immunother Cancer. (2021) 9(10):e003464. doi: 10.1136/jitc-2021-003464 34599020 PMC8488710

[48] Hubert P Roncarati P Demoulin S Pilard C Ancion M Reynders C. Extracellular HMGB1 blockade inhibits tumor growth through profoundly remodeling immune microenvironment and enhances checkpoint inhibitor-based immunotherapy. J Immunother Cancer. (2021) 9(3):e001966. doi: 10.1136/jitc-2020-001966 33712445 PMC7959241

[49] Pan Y Lu F Fei Q Yu X Xiong P Yu X. Single-cell RNA sequencing reveals compartmental remodeling of tumor-infiltrating immune cells induced by anti-CD47 targeting in pancreatic cancer. J Hematol Oncol. (2019) 12:124. doi: 10.1186/s13045-019-0822-6 31771616 PMC6880569

[50] Wu ZB Cai L Lin SJ Xiong ZK Lu JL Mao Y. High-mobility group box 2 is associated with prognosis of glioblastoma by promoting cell viability, invasion, and chemotherapeutic resistance. Neuro Oncol. (2013) 15:1264–75. doi: 10.1093/neuonc/not078 PMC3748920 23828241

[51] Chen YZ Meng ZS Xiang ZL. HMGB2 drives tumor progression and shapes the immunosuppressive microenvironment in hepatocellular carcinoma: insights from multi-omics analysis. Front Immunol. (2024) 15:1415435. doi: 10.3389/fimmu.2024.1415435 39247201 PMC11380137

[52] Takeda T Izumi H Kitada S Uramoto H Tasaki T Zhi L. The combination of a nuclear HMGB1-positive and HMGB2-negative expression is potentially associated with a shortened survival in patients with pancreatic ductal adenocarcinoma. Tumour Biol. (2014) 35:10555–69. doi: 10.1007/s13277-014-2328-8 25060178

[53] Cui G Cai F Ding Z Gao L. HMGB2 promotes the Malignancy of human gastric cancer and indicates poor survival outcome. Hum Pathol. (2019) 84:133–41. doi: 10.1016/j.humpath.2018.09.017 30296520

[54] Kang G Jiao Y Pan P Fan H Li Q Li X. α5-nAChR/STAT3/CD47 axis contributed to nicotine-related lung adenocarcinoma progression and immune escape. Carcinogenesis. (2023) 44:773–84. doi: 10.1093/carcin/bgad061 37681453

[55] Liu X Pu Y Cron K Deng L Kline J Frazier WA. CD47 blockade triggers T cell-mediated destruction of immunogenic tumors. Nat Med. (2015) 21:1209–15. doi: 10.1038/nm.3931 PMC4598283 26322579

[56] Peluso MO Adam A Armet CM Zhang L O'Connor RW Lee BH. The Fully human anti-CD47 antibody SRF231 exerts dual-mechanism antitumor activity via engagement of the activating receptor CD32a. J Immunother Cancer. (2020) 8(1):e000413. doi: 10.1136/jitc-2019-000413 32345627 PMC7213910

[57] Chao MP Jaiswal S Weissman-Tsukamoto R Alizadeh AA Gentles AJ Volkmer J. Calreticulin is the dominant pro-phagocytic signal on multiple human cancers and is counterbalanced by CD47. Sci Transl Med. (2010) 2:63ra94. doi: 10.1126/scitranslmed.3001375 PMC4126904 21178137

[58] Chen J Zheng DX Yu XJ Sun HW Xu YT Zhang YJ. Macrophages induce CD47 upregulation via IL-6 and correlate with poor survival in hepatocellular carcinoma patients. Oncoimmunology. (2019) 8:e1652540. doi: 10.1080/2162402X.2019.1652540 31646099 PMC6791434

[59] Rastgoo N Wu J Liu A Pourabdollah M Atenafu EG Reece D. Targeting CD47/TNFAIP8 by miR-155 overcomes drug resistance and inhibits tumor growth through induction of phagocytosis and apoptosis in multiple myeloma. Haematologica. (2020) 105:2813–23. doi: 10.3324/haematol.2019.227579 PMC7716364 33256380

[60] Shi Z Tu J Ying Y Diao Y Zhang P Liao S. CC chemokine ligand-2: A promising target for overcoming anticancer drug resistance. Cancers (Basel). (2022) 14(17):4251. doi: 10.3390/cancers14174251 36077785 PMC9454502

[61] Razaghi A Heimann K Schaeffer PM Gibson SB. Negative regulators of cell death pathways in cancer: perspective on biomarkers and targeted therapies. Apoptosis. (2018) 23:93–112. doi: 10.1007/s10495-018-1440-4 29322476

[62] Weiskopf K. Cancer immunotherapy targeting the CD47/SIRPα axis. Eur J Cancer. (2017) 76:100–9. doi: 10.1016/j.ejca.2017.02.013 28286286

[63] Strati P Hawkes E Ghosh N M J Tuscano Q Chu Q. Interim results from the first clinical study of CC-95251, an anti-signal regulatory protein-alpha (SIRPα) antibody, in combination with rituximab in patients with relapsed and/or refractory non-hodgkin lymphoma (R/R NHL). Blood. (2021) 138:2493. doi: 10.1182/blood-2021-147292

