# Supplementary figures and images for: Pan-cancer analysis of phagocytosis regulators in female-specific cancers: a focus on HMGB2
Source: Front Immunol. 2025 May 6;16:1565924. doi: 10.3389/fimmu.2025.1565924 (PMC12089064; doi:10.3389/fimmu.2025.1565924)

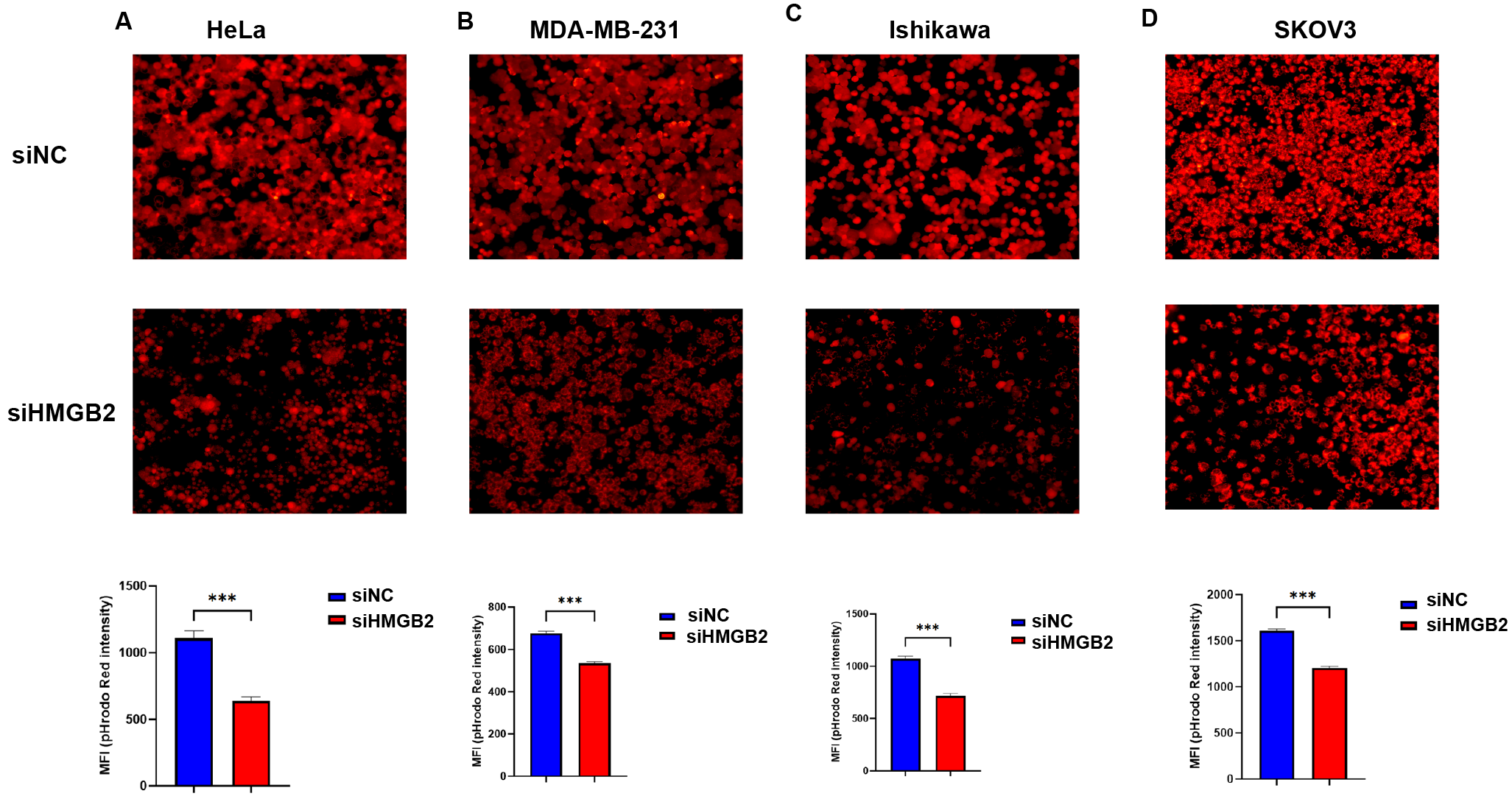

Supplement: Supplementary Figure 1 — Phagocytic activity of RAW264.7 macrophages towards different tumor cell types following HMGB2 knockdown. A. RAW264.7 cells with HMGB2 knockdown (siHMGB2) exhibited reduced phagocytic activity compared to the control (siNC) in the HeLa cell. B. RAW264.7 cells with HMGB2 knockdown (siHMGB2) exhibited reduced phagocytic activity compared to the control (siNC) in the MDA-MB-231 cell. C. RAW264.7 cells with HMGB2 knockdown (siHMGB2) exhibited reduced phagocytic activity compared to the control (siNC) in the Ishikawa cell. D. RAW264.7 cells with HMGB2 knockdown (siHMGB2) exhibited reduced phagocytic activity compared to the control (siNC) in the SKOV3 cell. ***P < 0.001. [file Image1.tif]

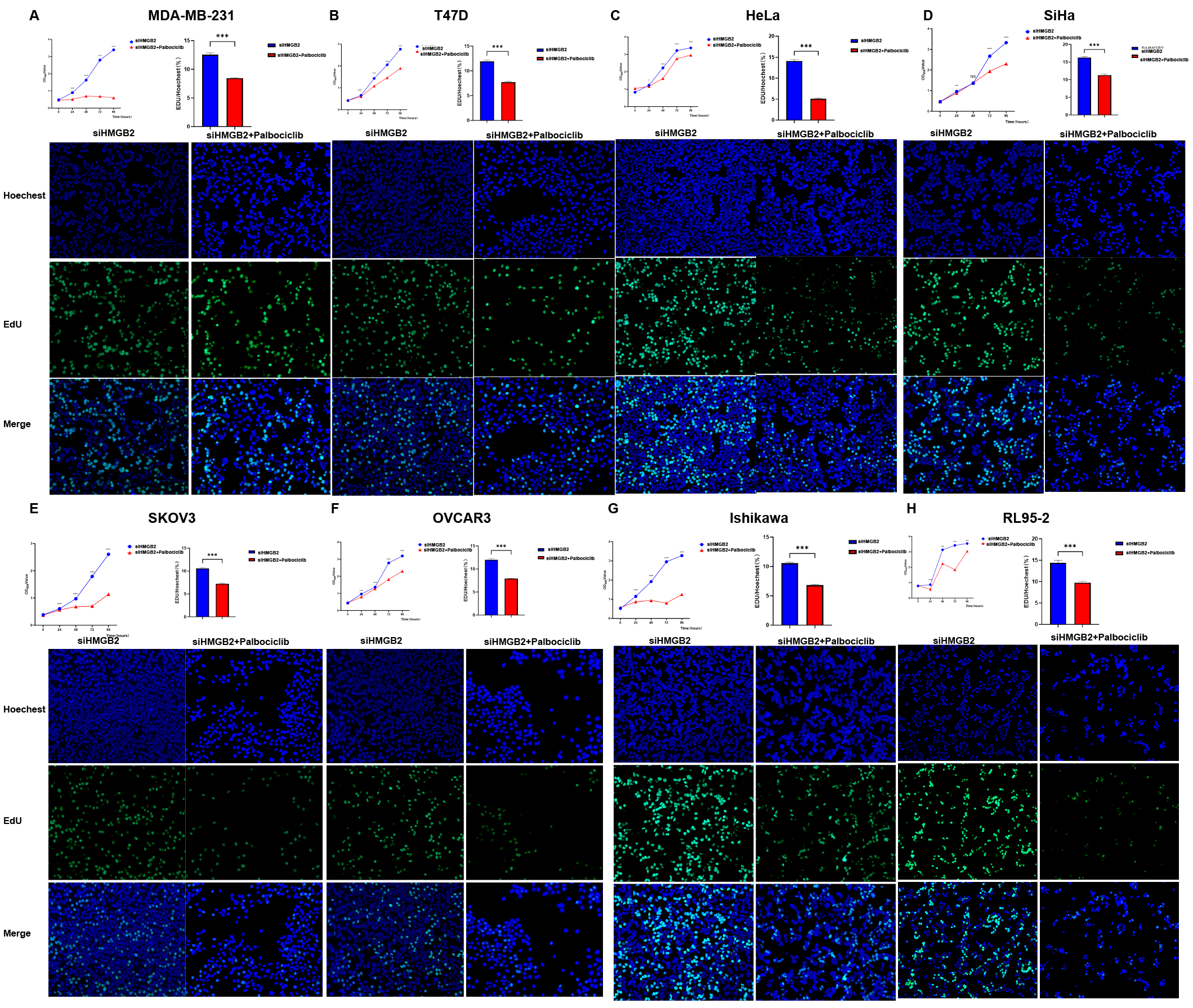

Supplement: Supplementary Figure 2 — Effect of HMGB2 knockdown and Palbociclib treatment on tumor cell proliferation, as assessed by EdU fluorescence staining. (A) Knockdown of HMGB2 in MDA-MB-231 cells followed by Palbociclib treatment significantly suppressed cell proliferation, as evidenced by CCK8 and EdU assays. (B) Knockdown of HMGB2 in T-47D cells followed by Palbociclib treatment significantly suppressed cell proliferation, as evidenced by CCK8 and EdU assays. (C) Knockdown of HMGB2 in HeLa cells followed by Palbociclib treatment significantly suppressed cell proliferation, as evidenced by CCK8 and EdU assays. (D) Knockdown of HMGB2 in SiHa cells followed by Palbociclib treatment significantly suppressed cell proliferation, as evidenced by CCK8 and EdU assays. (E) Knockdown of HMGB2 in SK-OV-3 cells followed by Palbociclib treatment significantly suppressed cell proliferation, as evidenced by CCK8 and EdU assays. (F) Knockdown of HMGB2 in OVCAR3 cells followed by Palbociclib treatment significantly suppressed cell proliferation, as evidenced by CCK8 and EdU assays. (G) Knockdown of HMGB2 in Ishikawa cells followed by Palbociclib treatment significantly suppressed cell proliferation, as evidenced by CCK8 and EdU assays. (H) Knockdown of HMGB2 in RL95–2 cells followed by Palbociclib treatment significantly suppressed cell proliferation, as evidenced by CCK8 and EdU assays. NS>0.05,*P < 0.001, **P < 0.001, ***P < 0.001. [file Image2.tif]
